# Molecular basis for the increased affinity of an RNA recognition motif with re-engineered specificity: A molecular dynamics and enhanced sampling simulations study

**DOI:** 10.1371/journal.pcbi.1006642

**Published:** 2018-12-06

**Authors:** Anna Bochicchio, Miroslav Krepl, Fan Yang, Gabriele Varani, Jiri Sponer, Paolo Carloni

**Affiliations:** 1 Computational Biomedicine, Institute for Advanced Simulation IAS-5 and Institute of Neuroscience and Medicine INM-9, Forschungszentrum Jülich, Jülich, Germany; 2 Institute of Biophysics of the Czech Academy of Sciences, Brno, Czech Republic; 3 Department of Chemistry, University of Washington, Seattle, Washington, United States of America; 4 Regional Centre of Advanced Technologies and Materials, Department of Physical Chemistry, Faculty of Science, Palacky University Olomouc, Olomouc, Czech Republic; 5 JARA-HPC, Jülich Supercomputing Centre, Forschungszentrum Jülich GmbH, Jülich, Germany; University of Missouri, UNITED STATES

## Abstract

The RNA recognition motif (RRM) is the most common RNA binding domain across eukaryotic proteins. It is therefore of great value to engineer its specificity to target RNAs of arbitrary sequence. This was recently achieved for the RRM in Rbfox protein, where four mutations R118D, E147R, N151S, and E152T were designed to target the precursor to the oncogenic miRNA 21. Here, we used a variety of molecular dynamics-based approaches to predict specific interactions at the binding interface. Overall, we have run approximately 50 microseconds of enhanced sampling and plain molecular dynamics simulations on the engineered complex as well as on the wild-type Rbfox**·**pre-miRNA 20b from which the mutated systems were designed. Comparison with the available NMR data on the wild type molecules (protein, RNA, and their complex) served to establish the accuracy of the calculations.

Free energy calculations suggest that further improvements in affinity and selectivity are achieved by the S151T replacement.

## Author summary

RNA is an outstanding target for oncological intervention. Engineering the most common RNA binding motif in human proteins (called RRM) so as to bind to a specific RNA has an enormous pharmacological potential. Yet, it is highly non trivial to design RRM-bearing protein variants with RNA selectivity and affinity sufficiently high for clinical applications. Here we present an extensive molecular simulation study which shed light on the exquisite molecular recognition of the empirically-engineered complex between the RRM-bearing protein Rbfox and its RNA target pre-miR21. The simulations allow predicting a variant, the S151T, which may lead to further enhancement of selectivity and affinity for pre-miR21.

## Introduction

The RNA recognition motif (RRM) is the largest family of eukaryotic RNA-binding proteins [1], involved in virtually all post-transcriptional regulatory events [2]. RRMs bind a wide-range of single-stranded RNAs [3], stem-loops and other RNA structures [2, 4–6]. Therefore, engineering RRM binding interfaces to target specific RNAs may create widely applicable tools for regulating gene expression [7, 8]. Yet, a variety of factors have hampered such efforts, including the complexities of the protein-RNA interactions, a poor understanding of the structural and biophysical basis for specificity, and the idiosyncratic way in which various RRM domains bind to RNA [9, 10].

**Fig 1:**
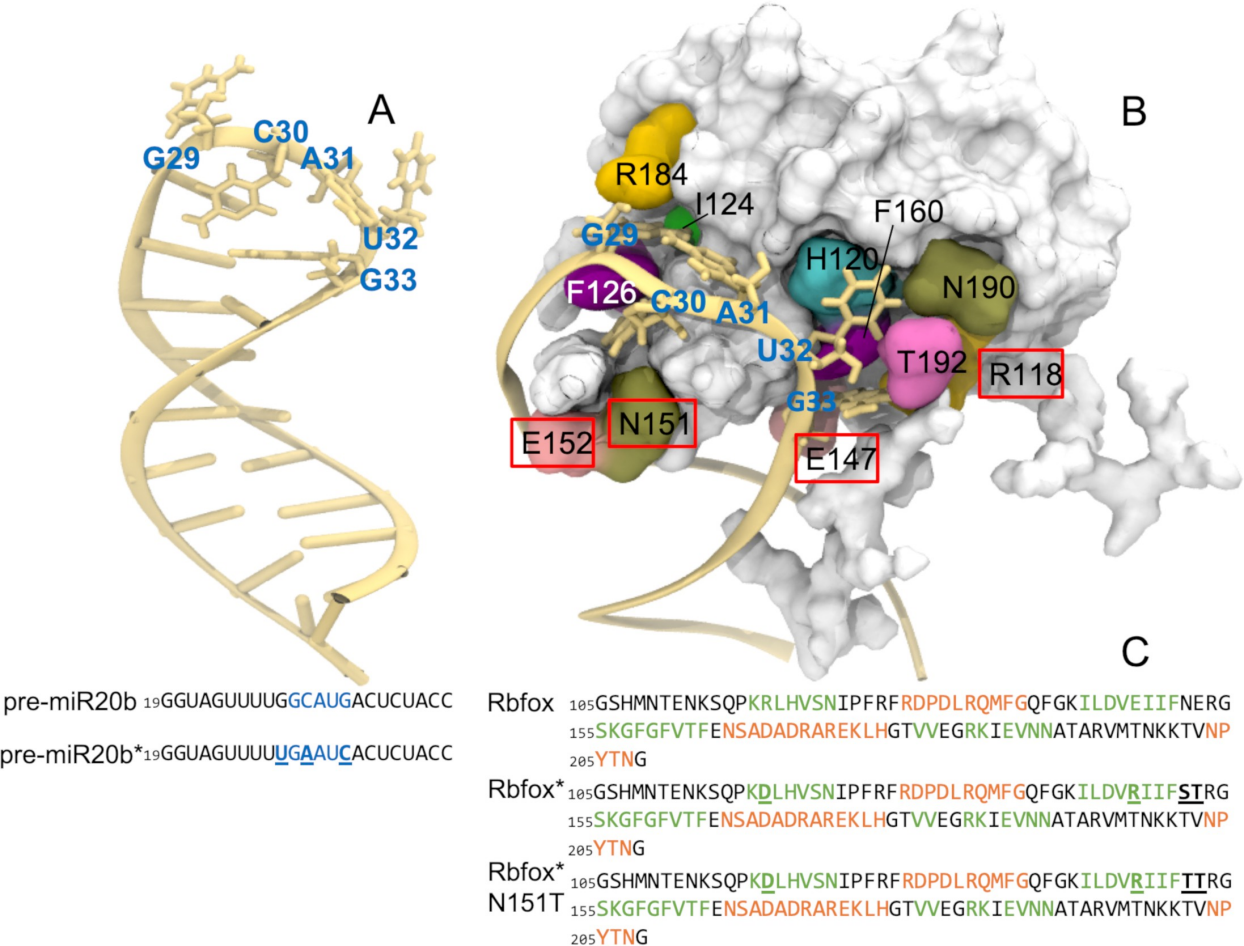
(A) NMR structure of pre-miR20b RNA (pdb 2n7x). (B) NMR structure of the Rbfox•pre-miR20b complex (pdb 2n82). The residues at the binding interface are highlighted. The amino acids labelled with a red square correspond to the mutated residues in Rbfox* and in Rbfox* S151T. (C) Left: nucleotide sequence of the pre-miR20b and of the mutant pre-miR20b*. Right: amino acids sequence of the Rbfox, Rbfox* and Rbfox* S151T mutants. Highlighted in green and in orange are the amino acids corresponding to β strands and α helices, respectively. The mutated nucleotides and amino acids are underlined.

Recently, some of us were able to engineer the conserved RRM domain of the human Rbfox protein by modulating its specificity for a target RNA [8]. The protein is part of a small family of tissue-specific alternative splicing regulators. It was chosen for its ability to bind with high sequence specificity and affinity -in the low nM range- to the r-GCAUG sequence in specific RNAs. These are the single-stranded RNAs and the hairpin microRNA precursors that code for miR107 and miR20b (referred to as pre-miR20b, hereafter, see Fig 1)[3, 6]. The r-G_29_AAUC_33_ sequence in the terminal loop of the chosen RNA target, the oncogenic precursor miRNA 21 (pre-miR21) [11], bears two nucleotide changes (at positions 30 and 33) from the r-GCAUG sequence. These mutations are sufficient to nearly abolish Rbfox binding [8]. The successfully engineered R118D-E147R-N151S-E152T quadruple mutant (Rbfox* hereafter, Fig 1) binds tightly to the pre-miR21 terminal loop sequence (*K*_d_ ~ 13 nM) [8], but also to pre-miR20b, with a dissociation constant only ~10 fold higher (*K*_d_ ~ 150 nM) [8]. Further improvements in binding specificity could be facilitated by understanding of the structural dynamics of key interactions at the protein-RNA interface at atomic level of description.

Molecular Dynamics (MD) simulations in explicit solvent are a useful tool to dissect the nature of interactions and specificity in biomolecular complexes [12, 13], providing information beyond what can be obtained experimentally. In particular, MD nicely complements NMR experiments on RNA interactions with RRM class of binding domains [14–16] by providing insights into specific interactions that are not revealed by experiments.

In this manuscript, we report the use of molecular simulation approaches to predict the structural determinants of the Rbfox*•pre-miR21 complex. After performing standard simulations, we use free-energy calculations to investigate a new mutant (S151T Rbfox*) that is predicted to improve selectivity towards the pre-miR21 target RNA relative to Rbfox*. The accuracy of our simulations is established by a comparison with the available NMR data and structure of the Rbfox•pre-miR20b complex [6, 17].

## Results and discussion

As a first task **(i)**, we tested our computational setup by performing extensive MD simulations, extended to the microsecond timescale, on the Rbfox•pre-miR20b complex in explicit solvent. Note that the RNA hairpin loop is remodeled by the protein compared to the free RNA; in the complex the hairpin segment is larger to accommodate the protein [6]. The RNA/protein interface is stabilized by a number of intermolecular stacking interactions and hydrogen bonds, which provide tight sequence specificity for the nucleotide sequence–G_29_C_30_A_31_U_32_G_33_-. The MD results reproduce the available NMR structural data well and describe accurately the interactions at the binding interface. Comparison with simulations of the two isolated molecules (protein and RNA) suggests significant changes of the protein flexibility upon complex formation. Next **(ii),** we constructed a model of the binding interface of the Rbfox*•pre-miR21 complex by replacing G_28_, C_30_ and G_33_ with U, A and C, respectively, and by substituting R118, E147, N151 and E152 residues with D, R, S, and T, respectively. We performed a series of explicit-solvent simulations on the resulting model. To ensure adequate sampling of the conformational space of the mutated complex, we used an enhanced sampling method (Replica Exchange with solute scaling -REST2- [17] simulations). The results were consistent with affinity data.

(**iii**) To further cross-validate the simulations predictions on the Rbfox*•pre-miR21 binding interface, we performed two additional simulations on the Rbfox•pre-miR21 and Rbfox*•pre-miR20b systems. The molecular description of the interactions at the two binding interfaces is in qualitative agreement with the experimental binding affinity data.

Finally **(iv)**, we used the simulated model of the Rbfox*•pre-miR21 complex to design a mutant with predicted higher affinity and selectivity.

The studied protein-RNA complexes are characterized by a complex interplay between the sequence and structural dynamics. Therefore, quantitative analysis of the simulation trajectories is not trivial. To do so, we have employed a wide range of different descriptors to characterize the protein conformational dynamics and plasticity (RMSD and PAD [18]), RNA structural variation (*ε*RMSD [19] and structural parameters: torsion angles, base-pair and base-pair steps parameters), and to quantify the change in protein stability upon mutations and RNA binding (conformational entropy).

### Validation of simulation setup (Simulations 1–13 in Table 1)

**Table 1:** Simulations performed in this work.

Simulated systems and simulation numbers	Length
**Rbfox**	
**1**	1 μs
**Pre-miR20b**	
**2**	1 μs
**3**^a^	1 μs
**4**^a^	1 μs
**5**^a^	1 μs
**6**^a^	1 μs
**7**^a^	1 μs
**1 (χ**_**OL3**_**-CP-OPC)**^b^	1 μs
**2 (χ**_**OL3**_**-CP-OPC)**^b^	1 μs
**3 (χ**_**OL3**_**-CP-OPC)**^b^	1 μs
**Rbfox•pre-miR20b**	
**8**	1 μs
**9**^a^	1 μs
**10**^a^	1 μs
**11**^a^	1 μs
**12**^a^	1 μs
**13**^a^	1 μs
Rbfox*	
**14**	1 μs
Rbfox*•pre-miR20b*	
**15 (REST2 PS)**^c^	8 × 2 μs
**16 (REST2)**	16 × 1 μs
**17**	1 μs
**18**	1 μs
S151T Rbfox*•pre-miR20b*	
**19**	0.9 μs
**20**	0.5 μs
**21 (FE)**	10 × 100 × 200 ps^d^
**22 (FE)**	10 × 100 × 1 ns^d^
Rbfox•pre-miR20b*	
**23**	1 μs
Rbfox*•pre-miR20b	
**24**	1 μs

^a^NMR restrains applied for the first 120 ns

^b^ χ_OL3_-CP-OPC refers to MD simulations performed with the χ_OL3_ force field supplemented by Case modified phosphate’s oxygens van der Waals parameters [21] and using the OPC water model [22].

^c^REST2 *PS* refers to REST2 simulations with *partial scaling* of the solute atoms.

^d^Here, we report the number of independent runs, the number of forward and backward transformations, and the length of transformation, respectively.

Recent MD studies on diverse protein-RNA complexes provided indication that the standard equilibration protocols, usually sufficient to equilibrate isolated medium-size RNA or protein molecules, might be inadequate for simulations of protein-RNA complexes [20]. Therefore, we performed multiple microsecond-long simulations, and exploited the existing NMR information as restraints at the early stages of most simulations (details provided in Materials and Method section).

The properties described below are calculated on the unrestrained parts of the initially restrained simulations of the system (Table 1, sim. 3–7 and 9–13), and on the fully unrestrained runs (Table 1, sim. 2 and 8) for comparisons. The individual trajectories sampled a similar conformational space (S1 Fig). Consequently, the average structural and dynamic properties calculated over the entire MD ensemble (all trajectories merged) do not significantly differ from those determined over the individual trajectories.

### Rbfox protein flexibility in simulations (Simulations 1 and 8–13 in Table 1)

The root mean square deviation (RMSD) of the RRM domain (residues 117–193) in the Rbfox•pre-miR20b complex and in the free state in aqueous solution fluctuate around an average of 0.17 ± 0.02 nm, and 0.30 ± 0.02 nm, respectively, after only 200 ns (S2 Fig). This suggests that the systems are well equilibrated for most of the dynamic runs. These RMSD values are within the uncertainty of the NMR ensemble.

As expected [6], the protein RRM domain (residues 117–193) becomes generally more rigid upon RNA binding. Indeed, we calculate a substantial decrease in the *per residue* conformational entropy upon binding (S3 Fig).

**Fig 2:**
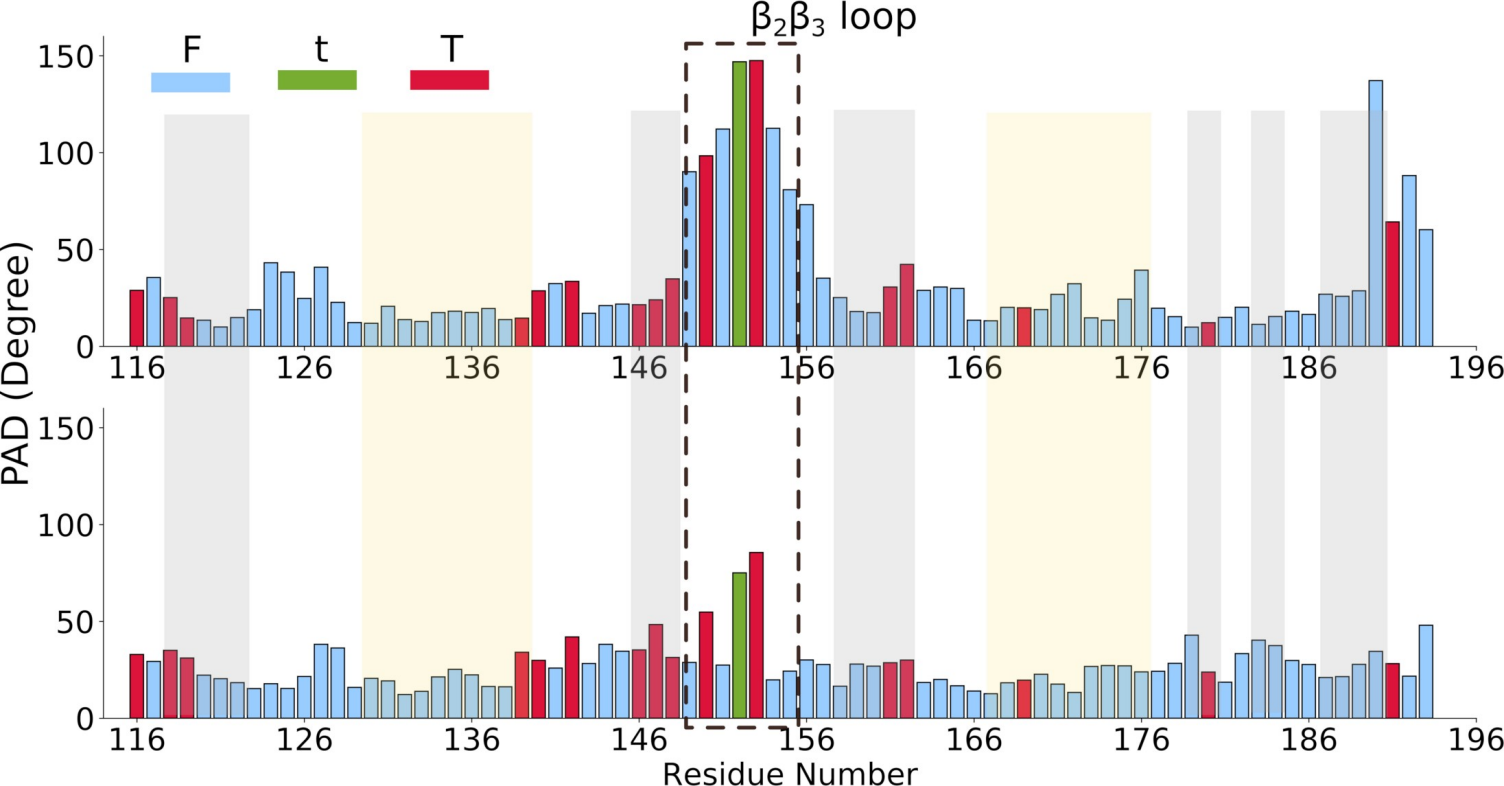
Fluctuations of the PAD angle in the simulations of free (top; see Table 1, sim. 1) and bound Rbfox protein (bottom; see Table 1, sim. 9–13). F indicates fluctuations; t short transitions and T long transitions. The structured regions of the protein are highlighted: the grey and yellow regions correspond to the β-strands and helices, respectively.

Finally, the backbone flexibility, described here in terms of the so-called Protein Angular dispersion for the Ramachandran angles (PAD) [18], is larger in the free state than in the Rbfox•pre-miR20b complex. The larger the PAD value, the more flexible the protein backbone. The same analysis also allows identification of conformational transitions of the backbone during simulations [18]. These involve residues belonging to the β_2_ and β_3_ strands and to the β_2_β_3_ loop. This region is inserted into pre-miR20b terminal loop and anchors the RNA to the protein surface (Fig 2) in a manner reminiscent of the structure of the U1A complex (PDB 1URN, [23]). However, unlike U1A, the Rbfox RRM binds much more strongly to a single stranded RNA compared to a stem-loop with the same binding sequence[6]. The pronounced flexibility of the β_2_β_3_ loop might not be optimal for binding to structured RNAs [24, 25].

### The RNA is structurally stable in simulations of the complex (Simulations 8–13) but not in isolation (Simulations 2–7 and 1–3 (χ_OL3_-CP-OPC) in Table 1).

The RNA conformational ensemble in the Rbfox•pre-miR20b complex simulations is compatible with that of the NMR ensemble (S5 Fig). In particular, the conformational flexibility of nucleotide U_27_, not bound to the protein, is relatively large both in the NMR ensemble and in simulations. It dominantly contributes to the observed relatively large εRMSD values (S4 Fig). If calculated on the loop nucleotides directly bound to the protein (G_28_GCAUG_33_), the average εRMSD value is only 0.76 ± 0.14.

The backbone torsion angles values of the loop region (nucleotides 28–33) and the stem base pair parameters remain in agreement with those calculated for the structures of the NMR ensemble (S6 Fig).

**Fig 3:**
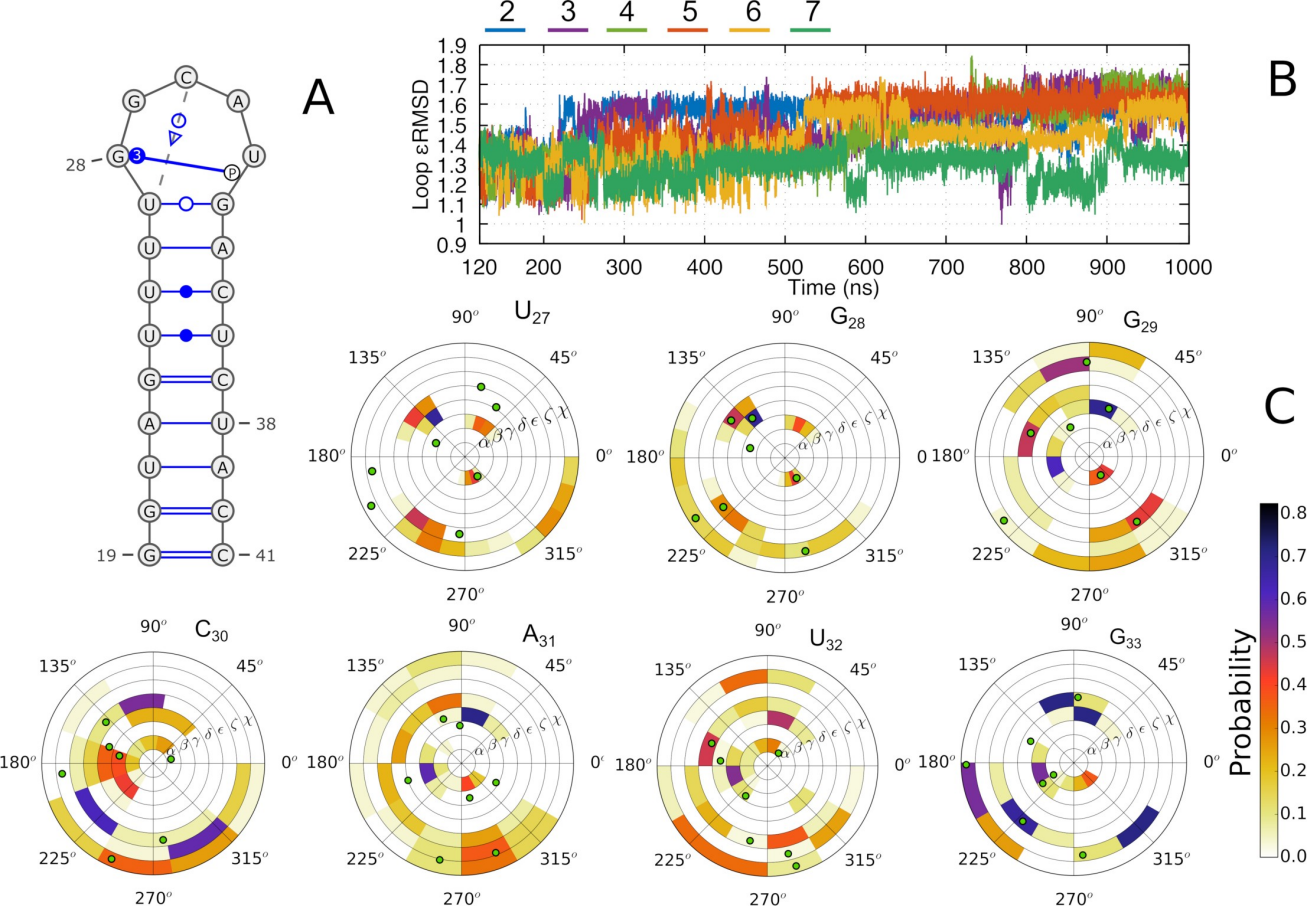
(A) 2D representation of the free pre-miR20b stem-loop structure. (B) Time development of the *ε*RMSD of the pre-miR20b loop (r-U_27_GGCAUG_33_) in the six χ_OL3_ MD simulations (Table 1, sim. 2–7). C. RNA backbone dihedral angle histograms calculated over the aggregated simulations. The green dots indicate the values of the angles in the lowest energy structure of the NMR ensemble 2n7x from which the simulations were started.

In contrast, the simulations show immediate and large-scale conformational changes within the apical loop of the free pre-miR20b RNA (we reiterate that the RNA structure in isolation differs significantly from the complex). Unsatisfactory behavior of simulations of RNA hairpin loops has been widely analysed in literature [13, 26]. It is ascribed to accumulation of various inaccuracies in the force fields, such as an overstabilization of non-native base-phosphate and/or sugar-phosphate interactions, underestimated stability of the hydrogen bonding interaction in base pairing and various difficulties in describing the sugar-phosphate backbone substates [13]. Therefore, the description of the apical loop of the free pre-miR20b RNA can be expected to be less accurate than that of the RNA in complex with the protein. Indeed, in all simulations of the free pre-miR20b RNA (Table 1, sim. 2–7), performed with the standard AMBER force field (χ_OL3_; described in Methods), the U_27_GGCAU_32_ loop is rearranged, and the original NMR conformation is never recovered afterwards (Fig 3, S7 Fig). Note that in the NMR structure, the loop is rigidly ordered and characterized by a U_27_-G_28_ stacking interaction and a G_28_-U_32_ type 3 base-phosphate (3BPh) interaction [27] (Fig 3).

To illustrate the changes in the loop conformations, we show the overlap of frames from each simulation and the NMR starting structure in S7 Fig. We observe high mobility of the G_29_ base, which flips around its χ torsion from *anti* to *syn* to stack with the G_28_ base (Fig 3), followed by the loss of the G_28_-U_32_ 3BPh interaction. The C_30_ base is either bulged out or forms stacking interactions with A_31_. The high mobility of C_30_ and U_32_ results in a significant distortion of the backbone torsion angles (Fig 3). Due to our inability to reproduce the NMR structure of the isolated RNA hairpin, we did not attempt any simulations of the isolated RNA starting from its conformation seen in the RNA/protein complex.

The pronounced flexibility of the terminal loop, however, does not affect the pre-miR20b stem dynamics, as shown by a comparison between the base pair and base–pair steps parameters of the simulated and NMR-solved ensembles of structures (S8 Fig).

Likewise, the inability to reproduce the experimental conformation of the apical loop in simulations of the free pre-miR20b RNA should not affect our investigations of the protein-RNA complex where the loop is sufficiently stabilized by the protein, in addition, the splayed RNA conformation is likely less strained than in the isolated hairpin loop as suggested by the experimental data [6].

It has been demonstrated that using the revised van der Waals phosphate’s oxygen parameters reported in ref. [21], along with the 3-charge, 4-point OPC water model [22] partially improves simulations of RNA tetranucleotides by stabilizing the native A-form like conformations [28, 29]; this protocol is referred as χ_OL3_-CP-OPC force-field combination, hereafter. However, for the pre-miR20b loop, the χ_OL3_-CP-OPC force field-based simulations did not achieve better accuracy than those based on the parent χ_OL3_ force field (Table 1, sim. 1–3 (χ_OL3_-CP-OPC)); a similar unsatisfactory outcome was reported also for other hairpin-loop systems investigated recently [26]. Indeed, the pre-miR20b χ_OL3_-CP-OPC simulations showed *syn/anti* nucleobase flips and alternative stacking conformations for all the terminal loop nucleotides (S6 Fig). Since no improvement was detected with this protocol, we did not attempt χ_OL3_-CP-OPC simulations of the protein-RNA complexes. The use of the modified phosphate parameters, while improving the A-form single-strand simulations, might destabilize for example some native BPh interactions in folded RNAs. Note that simulations of free RNA hairpin loops remain a fundamental challenge for all currently available RNA force fields [13][30].

Analysis of average ion occupancies [31] revealed an average local concentration of approximately 1 M sodium near the U_27_/G_33_ base pair in simulations of the free RNA. This is a significantly elevated ion concentration, well above the bulk value. Here, the Na^+^ ions interact with the (U_27_) O4 atom with a residency time of few ns, at a distance of ~ 0.25 nm (S9 Fig). This ion-binding site is completely absent in the complex structure since the U_27_/G_33_ base pair is disrupted by protein binding. Instead, in the complex, the Na^+^ concentration is strongly localized at the dinucleotide step 34–35 (S9 Fig).

### The RNA/protein interface

**Fig 4:**
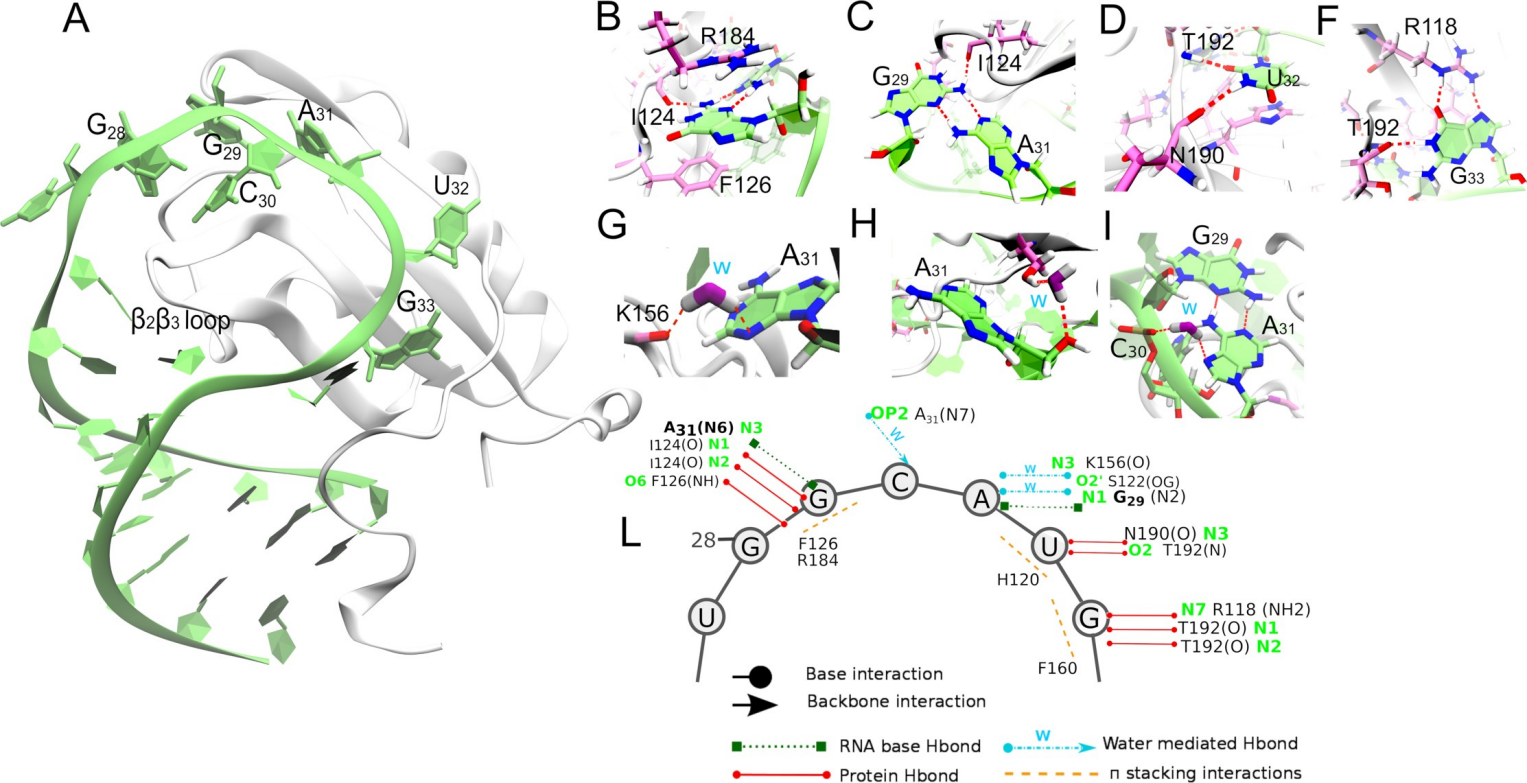
(A) Bird’s-eye view of the Rbfox•pre-miR20b complex. (B)-(H) Close-up views of the interactions observed at the binding interface during MD simulations. The H-bonds are indicated by dotted red lines. The depicted snapshots belong to the representative structures of the 20 clusters (“MD-adapted structure ensemble”), which have the highest agreement with NMR NOE data. (I) Scheme of the interactions. Circle and arrowheads depict interaction with RNA bases or phosphate groups, respectively.

The interface structure sampled in MD simulations is similar to that of the NMR-resolved ensemble (Fig 4). In particular, G_29_ stacks with F126 and R184. The G_29_/R184 stacking interaction is always observed in the simulations (S10 Fig) even though it is absent in some frames of the NMR ensemble. The network of interactions is further stabilized by a bifurcated hydrogen bond involving the I124 backbone and the G_29_ base (Table 2). As in the NMR ensemble, G_29_ and A_31_ form a *trans* Watson Crick/Shallow groove (tWS) base-pair [32] in the simulations. The G_29_/A_31_ interaction is further stabilized by a water molecule, coordinated by the A_31_ N7 atom and C_30_ phosphate group (Fig 4). The adenine base forms water-mediated hydrogen bonds with the S122 and K156 side chains. The residence time of water molecules in these interactions are of a few tens of ns, sensibly longer than the common time-scale (50–500 ps) of short-residency hydration sites around RNA molecules [33–35]. These results are fully consistent with the earlier report of structured hydration sites in the simulations of the Rbfox RRM in complex with single-stranded RNA [15].

**Table 2:** Hydrogen bonds at the binding interface of the Rbfox•pre-miR20b complex observed during the MD simulations (Table 1, sim. 8–13). The average donor-acceptor distances and angles are calculated over the entire simulation ensemble for the trajectory frames in which the individual H-bonds are observed. The interactions are further characterized by their occupancy in the individual simulation trajectories 8–13.

Acceptor	Donor	Distance (nm)	Angle (°)	8	9	10	11	12	13	NMR Ensemble
G_29_ (O6)	F126 (N)	0.31 ± 0.02	152 ± 1	60%	83%	72%	72%	72%	69%	75%
I124 (O)	G_29_ (N1)	0.28 ± 0.01	148 ± 1	85%	96%	96%	96%	92%	96%	100%
I124 (O)	G_29_ (N2)	0.29 ± 0.02	147 ± 2	90%	89%	88%	89%	90%	88%	100%
G_29_ (N3)	A_31_ (N6)	0.31 ± 0.02	158 ± 1	70%	75%	74%	69%	73%	74%	90%
A_31_ (N1)	G_29_ (N2)	0.30 ± 0.02	166 ± 2	95%	99%	98%	98%	99%	98%	90%
N190 (O)	U_32_ (N3)	0.31 ± 0.02	162 ± 1	89%	93%	95%	96%	95%	95%	100%
U_32_ (O2)	T192 (N)	0.31 ± 0.01	159 ± 1	90%	94%	95%	94%	95%	95%	100%
G_33_ (N7)	R118 (NH2)	0.29 ± 0.02	161 ± 1	79%	94%	95%	94%	95%	94%	40%
T192 (O)	G_33_ (N1)	0.28 ± 0.01	155 ± 2	70%	96%	98%	97%	67%	65%	90%
T192 (O)	G_33_ (N2)	0.28 ± 0.02	150 ± 1	73%	69%	74%	96%	68%	95%	100%

In our simulations, the U_32_ base forms stacking interactions with H120 (S10 Fig) and hydrogen bonds with the backbone of N190 and T192 (Table 2, Fig 4). G_33_ base is in *syn* conformation and forms stacking interactions with F160 (Fig 4, S10 Fig) and hydrogen bonds with T192 backbone and R118 side chain (Table 2, Fig 4). The latter interaction, always observed with good hydrogen bond geometry (Table 2) in the simulations, is present in eight conformers of the NMR ensemble, while in the other twelve the residue is solvent-exposed, perhaps due to insufficiently clear experimental information. Notably, the R118 side chain forms a very similar hydrogen-bonding interaction in the NMR structure of the Rbfox-RRM bound to the single-stranded RNA r-U_1_G_2_C_3_A_4_U_5_G_6_U_7_ [3].

Next, we present several comparisons with the primary NMR data, which is a very stringent way to judge the accuracy of simulations [14].

On average, the back-calculated Chemical Shifts (CSs) for ^13^C and ^1^H atoms of free and bound pre-miR20b, along with the ^13^C’, ^13^C_a_, ^13^C_b_, and ^15^N atoms of Rbfox in its complex with pre-miR20b are, within the accuracy and the limits of the empirical methods (LARMOR^d^ [36] and SHIFTX+ [37]) used for the predictions (S11 Fig), in fair agreement with experimental observations.

The agreement between observed and calculated chemical shifts is only fair because of a variety of reasons. These include the fact that SHIFTX+ is expected not to be able to accurately predict shifts of residues in close proximity to RNA. Indeed, the characteristic ring current and charge for non-protein like molecules are not included in the SHIFTX2 parameterization [38]. Larmor^D^ suffers from the same drawback as it was parameterized by excluding RNA’ structures in complex with proteins or other ligands in its training data set [36]. Moreover, the apparent agreement (S11 Fig, Pearson correlation coefficients R = 0.99 for ^13^C and R = 0.97 for ^1^H) with the measured chemical shifts for the free pre-miR20b RNA (S11 Fig), which shows large conformational changes in simulations, suggest that Larmor^D^ sensitivity to structural changes might be limited.

However, the SHIFTX+ predictions were still sensitive to spurious motions of F150 and F158 as shown by the calculated distributions of the N nuclei, which are characterized by multiple peaks (S13 Fig). This might be related to temporary flips of the χ_1_ dihedral angles from gauche(+) to gauche(-) during the simulations (S13 Fig). A similar pronounced flexibility for phenylalanine and tyrosine side chains was observed also in our previous simulations using the ff14SB force field [15, 16]. This potentially erroneous behavior can be related to the energy barrier for side chain rotation around the χ_2_ angle [15], which might be too low in the employed force field.

The chemical shift predictions for the C_a_ nuclei of residues V146, E147, and V151, located on β_2_ strand, deviate from the experimental values beyond two standard deviations (S12 Fig). In this case, the divergence reflects the dependence of the carbon shifts on backbone φ and ψ angles, for which we already described enhanced fluctuations during the simulations (Fig 2).

None of the back-calculated CSs of the bound RNA show significant differences compared to the experimental values (S15 Fig).

On average, ~84% of the NOE upper bounds are satisfied for the isolated protein and RNA and only ~3% of the violated NOE have violations greater than 0.05 nm (S1 Table). Not unexpectedly, in free RNA, the larger NOE violations are typically localized to the G_28_, G_29_ and U_32_ nucleotides of the apical loop, which exhibit major conformational changes in the simulations (see above).

For the complex: on average, the percentage of satisfied NOE upper bounds is about 76%. Interestingly, the single trajectory generated without the initial application of the NMR restraints (13), shows the largest number of violations (Table 3). This indicates that the application of the experimental restraints in the early part of production trajectory leads to visibly better agreement with the inter-molecular NOEs in the subsequent unrestrained trajectories. However, this assessment should be taken with some caution, because it is made based on only a single unrestrained trajectory. The greatest distance violations (>0.3 nm) are observed for the inter-molecular NOEs involving amino acids F150 and F158 and nucleotides U_25_, and U_32_, possibly because of the relatively high flexibility of the two phenylalanine side chains noted above.

**Table 3:** Percentage of intra- and intermolecular NOE violations observed in the course of the simulations of the protein-RNA complex. Trajectory 8 (Table 1) has been obtained without applying the restraints in the initial stages of the simulation–see Methods.

Simulation	Intra RNA	Intra Protein	Inter Protein-RNA
**8**	19%	24%	48%
**9**	17%	22%	34%
**10**	18%	21%	32%
**11**	17%	22%	33%
**12**	19%	22%	32%
**13**	18%	21%	33%

Altogether, these analyses demonstrate that the MD-derived conformational ensemble of structures reproduces fairly well the experimentally sampled conformations for the Rbfox•pre-miR20b complex. These and previous [14] results suggest that our protocol can be employed to study the dynamic properties of the engineered Rbfox*•pre-miR20b* complex and to compare it with the wild type complex from which it was designed.

### The Rbfox*•pre-miR20b* complex

One of our main goals was to perform atomistic simulations of the Rbfox*•pre-miR20b* complex, for which the experimental structure is not available, and characterize the molecular interactions at its binding interface. The complex features R118D, E147R, N151S and E152T mutations on Rbfox, as well as G_28_U, C_30_A and G_33_C mutations in the pre-miR20b RNA. To achieve wide exploration of the engineered binding interface conformational space, we used enhanced sampling methods, and specifically Hamiltonian replica exchange (HREX) MD simulations. These have become a common way to elucidate conformational ensembles of proteins [39–41] and nucleic acids [42, 43]. HREX simulations should eliminate any bias caused by the initial building up of the mutated structure. A wealth of recipes for HREX has been proposed in the last years (among others, [39, 44–50]). One of the most successful approaches is the so-called replica exchange solute tempering in its REST2 variant [17], in which only the solute Hamiltonian is scaled. Being mainly interested in the properties of the Rbfox*•pre-miR20b* binding interface, we have used a promising cost-saving variant of REST2, where only this part of the solute is scaled (Methods, REST2 PS, Table 1, sim. 15). However, a standard REST2 simulation was also performed (Methods) for comparison (Table 1, sim. 16). A discussion of convergence issues of these types of simulations is offered in the SI.

**Fig 5:**
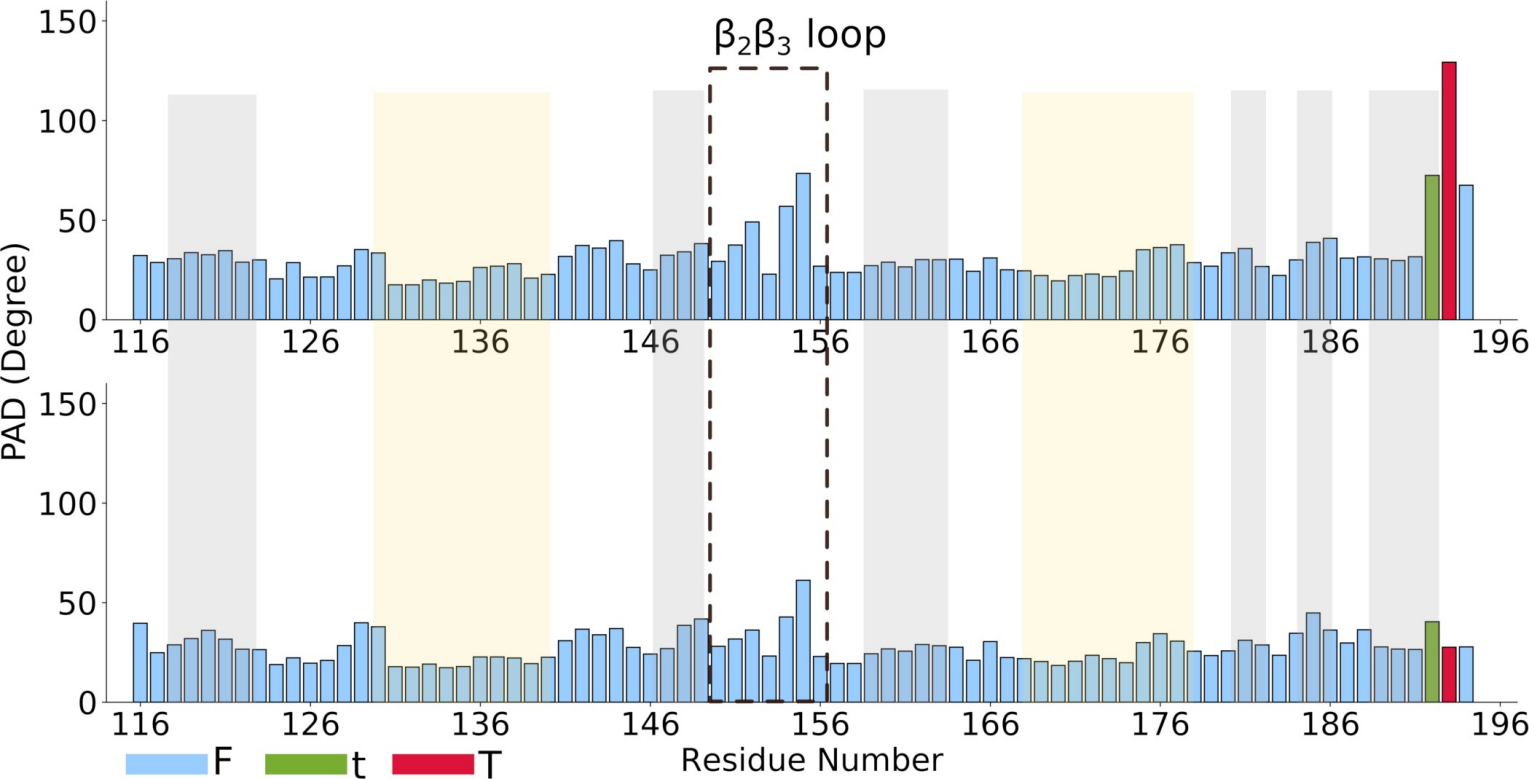
PAD and tag analyses for Rbfox* free (top) and bound to pre-miR20b* RNA (bottom). “F” indicates fluctuations; “t” short transitions and “T” long transitions. The secondary structure regions of the proteins are highlighted as in Fig 1.

Overall, the structure of the mutated complex remains very similar to that of the wild type, while the flexibility is reduced (Fig 5 and Table 4). Unlike in the wild-type, no backbone conformational transitions are observed for the β_2_β_3_ loop in the MD simulations of either the free (Table 1, sim. 14) or the bound (Table 1, sim. 17–18) Rbfox* (Fig 5).

**Table 4:** Conformational entropy differences associated with Rbfox* protein for residues belonging to the β_2_β_3_ loop. ΔS values are calculated as *S*_*RbfoxF*_*—S*_Rbfox*F_ (ΔS^a^) and *S*_Rbfox*F_*—S*_Rbfox*C_ (ΔS^b^), over simulations 1 (Rbfox free), 14 (Rbfox* free), and 17 (Rbfox* bound) as listed in Table 1. The subscripts F and C refer to the free and bound proteins, respectively. These values are obtained with very approximate methods, and they should be taken only for qualitative comparisons.

Residues	TΔS^a^ (kcal/mol)	TΔS^b^(kcal/mol)
F150	0.97 ± 0.04	0.57 ± 0.02
N151S	0.73 ± 0.02	0.16 ± 0.01
E152T	1.23 ± 0.01	0.13 ± 0.01
R153	1.22 ± 0.02	0.45 ± 0.03
G154	0.72 ± 0.01	0.3 ± 0.02

The results of this analysis are consistent with a considerable loss of per-residue conformational entropy of the Rbfox* β_2_β_3_ loop residues (Table 4 and S4 Fig). Upon RNA binding, the protein and the β_2_β_3_ loop become even less mobile. This is shown by a calculation of the so-called PAD values, which provide a measure of proteins’ backbone conformational flexibility and of the conformational entropy differences (Fig 5 and Table 4).

This increase in rigidity of the protein structure, and in particular of the loop—whose stiffening might provide a better steric fit for RNA binding [23]—might contribute to the 2-fold increase of binding affinity for E152T relative to the wild type protein [8].

**Fig 6:**
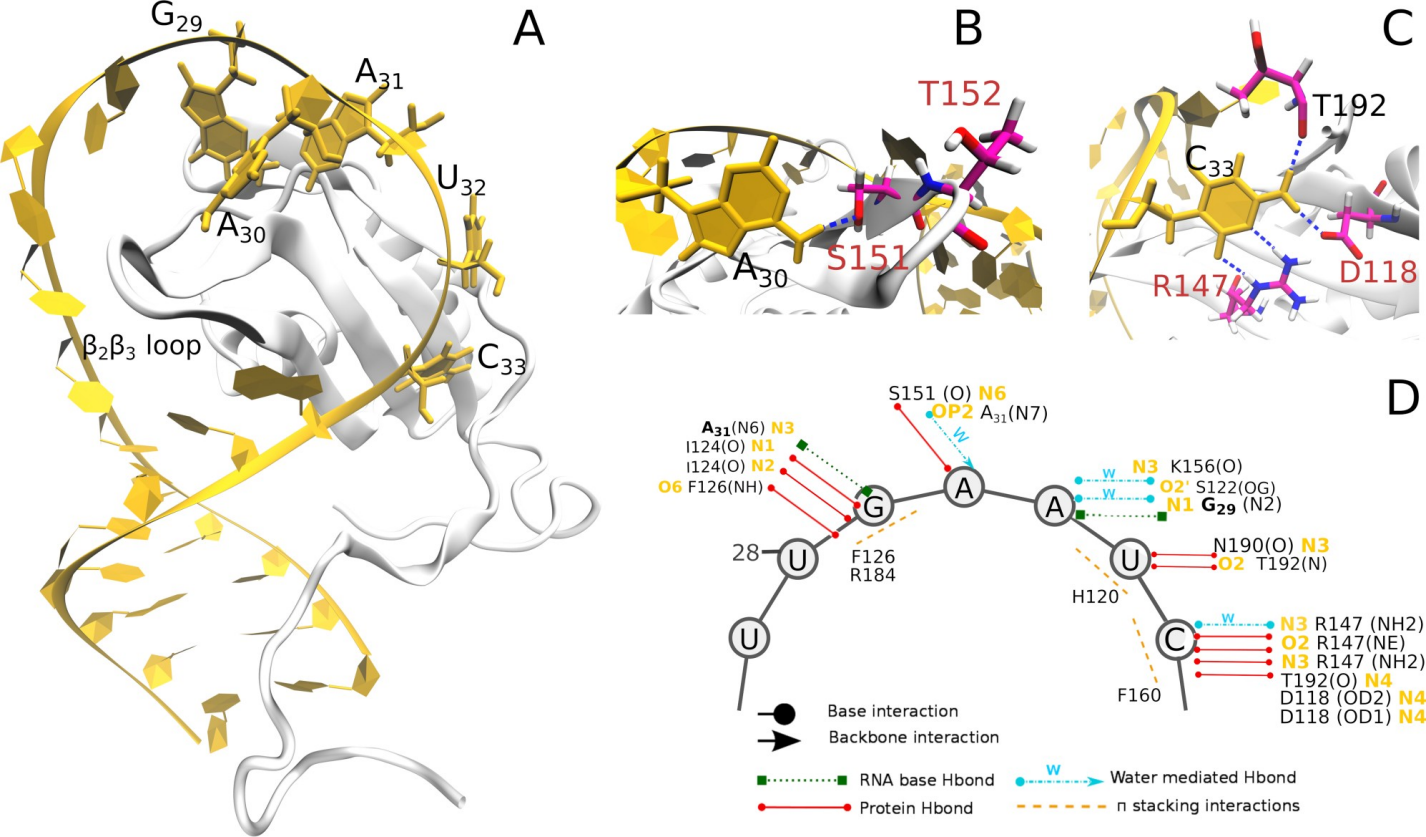
(A) Bird’s-eye view of the Rbfox*•pre-miR20b* complex. (B) and (C) Close-up views of the interactions established by the mutated residues with A_30_ and C_33_, respectively. (D) Scheme of the interactions between pre-miR20b* and Rbfox* observed in the MD simulations. Circle and arrowheads depict interaction with RNA bases or phosphate groups, respectively.

At the RNA/protein interface of the mutant, the hydrogen bonds, and stacking interactions of the G_29_, A_31_ and U_32_ bases (parts of the RNA/protein interface which are not mutated) observed in the wild-type simulations (Fig 4) are also preserved in the mutant complex (Fig 6, Table 5, S16 Fig). Interestingly, the water molecules coordinated by A_31_, S122 and K156 side chains as in the wild-type complex exhibit slow exchange with bulk solvent, with residence time of tens of ns (Figs 4 and 6). This leads us to suggest that these hydration sites could be indeed important in stabilizing the binding interface [15].

**Table 5:** Hydrogen bonds at the binding interface of the Rbfox*•pre-miR20b* complex during simulations. The average donor-acceptor distances and angles are calculated over the entire simulation ensemble for the trajectory frames in which the individual H-bonds are observed and the interactions are further characterized by their occupancy in the individual simulation trajectories. The trajectories are numbered as in Table 1.

Acceptor	Donor	Distance (nm)	Angle (degree)	15 (REST2 PS)	16 (REST2)	17	18
I124 (O)	G_29_ (N1)	0.29 ± 0.01	149 ± 1	96%	95%	96%	96%
I124 (O)	G_29_ (N2)	0.29 ± 0.02	148 ± 1	93%	95%	94%	94%
G_29_ (O6)	F126 (N)	0.31 ± 0.02	154 ± 2	70%	75%	72%	73%
G_29_ (N3)	A_31_ (N6)	0.31 ± 0.02	160 ± 1	75%	70%	74%	69%
A_31_ (N1)	G_29_ (N2)	0.30 ± 0.02	166 ± 2	99%	95%	98%	98%
N190 (O)	U_32_ (N3)	0.31 ± 0.02	162 ± 1	93%	94%	96%	96%
S151 (O)	A_30_ (N6)	0.29 ± 0.03	151 ± 1	28%	27%	30%	29%
U_32_ (O2)	T192 (N)	0.31 ± 0.01	154 ± 1	91%	90%	95%	90%
C_33_ (N3)	R147 (NH2)	0.29 ± 0.01	163 ± 1	99%	99%	97%	99%
C_33_ (O2)	R147 (NE)	0.29 ± 0.01	159 ± 2	98%	99%	97%	98%
T192 (O)	C_33_ (N4)	0.29 ± 0.01	160 ± 2	96%	97%	98%	97%
D118 (OD1/OD2)	C_33_ (N4)	0.29 ± 0.01	159 ± 1	99%	99%	97%	98%
S151 (OG)	G154 (O)	0.27 ± 0.01	159 ± 1	47%	45%	50%	48%

Overall, the simulations convincingly suggest that the system is able to structurally tolerate the mutations without altering the overall Rbfox*•pre-miR20b* binding mode.

A_30_ forms, about 30% of the time, a hydrogen bond with the S151 backbone (Table 5). This observation is consistent with the experimental data which shows only slight improvement of the mutant binding affinity relative to the wild type protein•pre-miR21 complex upon the N151S mutation [8]. Note that the S151 side chain mostly interacts with the N6 atom of A_30_ in simulations, instead of the N1 atom, as was originally suggested (8). When not present, the S151/A_30_ hydrogen bond is most often replaced by an intramolecular S151/G154 interaction (Table 5), which contributes to stabilizing the β_2_β_3_ loop.

C_33_, in the *anti* conformation (G_33_ in Rbfox•pre-miR20b complex was in *syn*), establishes either direct or water-mediated hydrogen bonds with the R147 guanidinium group (Fig 6). There are also hydrogen bonds with D118 and T192 side chains in most of the simulations (Table 5, Fig 6). This is consistent with the larger binding affinity gain of the R118D-E147R mutant for pre-miR21 relative to that of the wild type protein [8]. Indeed, these two mutations most significantly, increased the binding affinity of the mutant protein to pre-miR21 (by ~10^2^ fold) compared to the wild type protein•pre-miR21 complex [8]. Lastly, we note that the A_31_ forms an intermolecular stacking interaction with residue R153 within the β_2_β_3_ loop. This interaction is absent in the wild type complex. We suggest that the network of intermolecular interactions shown by our simulation is qualitatively consistent with the experimentally measured affinities and the rationale behind the design of the mutations [8].

### The Rbfox•pre-miR20b* and the Rbfox*•pre-miR20b complexes

To further investigate the accuracy of our predicted interactions at the engineered complex binding interface, we performed two 1-microsecond long simulations (Table 1, sim. 23 and 24). The first focuses on the Rbfox•pre-miR20b* complex and shows that the interface is solely maintained by the stacking interactions of G_29_ with F126 and R184 along with a hydrogen bond between I124 backbone and the G_29_ base (Table 6 and S18 Fig). This finding is qualitatively consistent with electrophoretic mobility shift assay experiments, which indicate an extremely weak binding for these substitutions [8]. The second simulation is carried out on the Rbfox*•pre-miR20b complex. The binding interface resulting from our simulation features equivalent hydrogen bonds and stacking interactions (Table 6) as observed in the wild type (Table 2 and Fig 4) and the Rbfox*•pre-miR20b* (Table 5 and Fig 6) complexes. These interactions involve the G_29_, A_31_ and U_32_ bases. The RNA does not interact with the protein’s β_2_β_3_ loop, and, in particular, with the mutated S151 and T152. Most notably, G_33_ maintains its *syn* conformation (as in the Rbfox•pre-miR20b complex) and forms a hydrogen bond with the mutated R147 side chain. The latter in turn forms a hydrogen bond with the mutated D118 (S19 Fig). Hence, the simulation suggests a strong compensatory effect upon amino acids substitution at this site as new interactions are formed to maintain complex stability. This may be consistent with the good binding affinity of the mutated Rbfox* protein features for the pre-miR20b terminal loop [8] (see Introduction).

**Table 6:** Hydrogen bonds at the binding interfaces of the Rbfox•pre-miR20b* and Rbfox*•pre-miR20b complexes in MD simulations. The average donor-acceptor distances and angles are calculated for the trajectory frames in which the individual H-bonds are observed and the interactions are further characterized by their occupancy.

Acceptor	Donor	Distance (nm)	Angle (degree)	Occupancy
Rbfox•pre-miR20b*				
I124 (O)	G_29_ (N1)	0.29 ± 0.02	150 ± 1	90%
I124 (O)	G_29_ (N2)	0.3 ± 0.01	146 ± 1	80%
G_29_ (O6)	F126 (N)	0.32 ± 0.01	153 ± 1	66%
Rbfox*•pre-miR20b				
I124 (O)	G_29_ (N1)	0.3 ± 0.01	149 ± 1	95%
I124 (O)	G_29_ (N2)	0.3 ± 0.02	147 ± 1	94%
G_29_ (O6)	F126 (N)	0.31 ± 0.02	151 ± 2	60%
G_29_ (N3)	A_31_ (N6)	0.30 ± 0.02	160 ± 1	74%
A_31_ (N1)	G_29_ (N2)	0.31 ± 0.02	167 ± 2	90%
N190 (O)	U_32_ (N3)	0.3 ± 0.02	162 ± 1	95%
U_32_ (O2)	T192 (N)	0.31 ± 0.01	157 ± 1	94%
G_33_ (N7)	R147 (NE)	0.29 ± 0.01	160 ± 1	75%
T192 (O)	G_33_ (N1)	0.29 ± 0.01	149 ± 1	76%
R147 (NH2)	D118 (OD2/OD1)	0.31 ± 0.02	150 ± 2	70%

These results, although based on single trajectories, further establish the simulations predictive power through their qualitative agreement with the experimental binding assays.

### *In silico* engineering a mutation with higher affinity and selectivity

Based on overall consistency between predictions and available experimental data, we sought to identify a mutation, which would further improve affinity and selectivity for the target pre-miR21b RNA. Specifically, our simulations show that the N151S substitution as suggested by the experiments [8] does not lead to significant interactions with the RNA, possibly because of the intrinsic flexibility of the protein β_2_β_3_ loop. We therefore reasoned that placing a bulkier group, such as Thr, in position 151 would be advantageous. Both S151 and T151 are capable of forming the same H-bonds with the N1 and/or the N6 atoms of A_30_. However, the bulkier side chain of T151 might influence the dynamics of the β_2_β_3_ loop. We therefore investigated the structure of S151T Rbfox*•pre-miR20b* by MD simulations (Table 1, sim. 19–20) and the change in affinity upon the S151T mutation by alchemical calculations using non-equilibrium approach (see Methods) [51]. The method has been successfully applied to a variety of protein mutants[51], and more recently, to protein–DNA–mutant complexes [52], providing accurate free energy estimates [52]. We refer to other works for a detailed comparison between the alchemical non-equilibrium and the equilibrium free energy calculations [53, 54].

**Fig 7:**
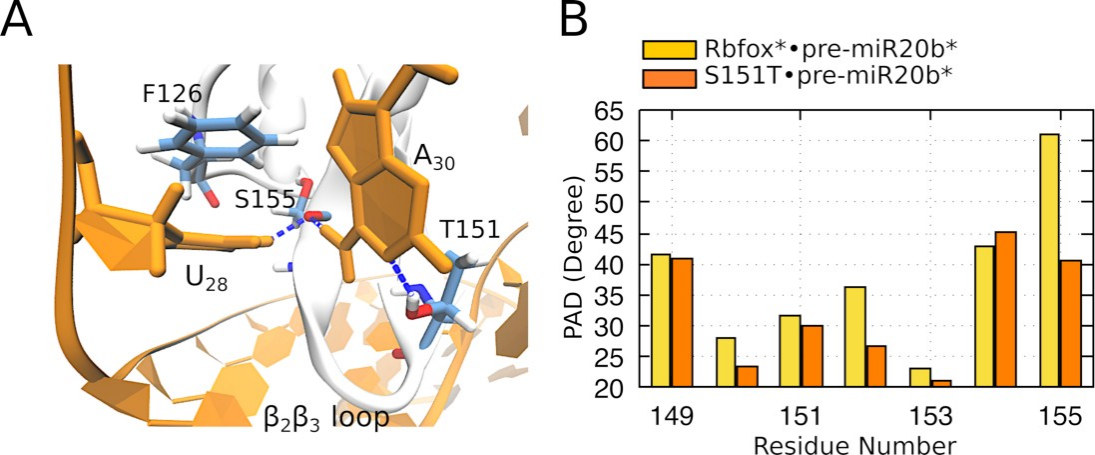
(A) Close up view on the interactions established by A_30_ with T151 and S155 and by U_28_ with S155 and F126. (B) Comparison between β_2_β_3_ loop and adjacent residues PAD values of the Rbfox* and S151T mutant in complex with pre-miR20b* RNA. The PAD values relate to the flexibility of the complex.

While the basic structure of the complex is overall unaffected, the A_30_(N6) forms hydrogen bond with the S155 backbone oxygen, while the A_30_(N1) forms a hydrogen bond with the T151 hydroxyl group (Fig 7). These interactions might decrease the flexibility of the β_2_β_3_ loop compared to the previous mutant (Fig 7), and lead to an indirect stabilization of the position of the U_28_ base, which is able to form a stable H-bond with the S155 backbone oxygen, and a stacking interaction with F126 (Fig 7). None of these interactions are observed in the Rbfox*•pre-miR20b* complex, where the U_28_ base is always solvent-exposed. Note that identical binding pattern for the U_28_ was also observed in earlier MD simulation studies of the wild type Rbfox complexed with a single-stranded RNA [14, 15]. The T151 thus might be better in overall accommodation of the pre-miR20b* RNA than the S151.

The free energy change associated with the S151T mutation, calculated using computational alchemy over two different simulation time windows (see Materials and Methods), is either -1.2 ± 0.3 kcal mol^-1^ or -1.3 ± 0.1 kcal mol^-1^. Hence, this estimation appears to be well converged and suggests that the mutation increases to a small, yet significant extent the affinity of the complex.

The presence of the U_28_ /protein interactions might also improve the selectivity of this mutant for the r-U_28_GAAUC_33_ sequence in pre-miR20b* RNA over r-G_28_GCAUG_33_ found in pre-miR20b. Indeed, in the wild type complex, G_28_ (equivalent to U_28_) exhibits pronounced flexibility in simulations and does not form any contact with the protein, ([6] and this work). Hence, our simulations suggest that the proposed mutation would alter the preference of the binding interface for the pre-miR20b* sequence over the pre-miR20b RNA, improving both the affinity and the selectivity of the engineered protein for the target pre-miR21 RNA.

## Conclusions

MD simulations of protein-RNA complexes remain somewhat limited by practical considerations of sampling (i.e. simulation time-scale) and inaccuracies resulting from force-field limitations [12, 20], yet they can supply important insight that often cannot be obtained by experiments, specifically on free-energy contributions and persistence of intermolecular contacts. The MD simulations in explicit solvent conducted here, covering overall about 50 microseconds of simulation data, including several state-of-the art simulation techniques and validated by their full consistency with experimental data, provide a detailed atomistic picture of the effect of mutations in the Rbfox*•pre-mir20b* interface. The simulations also suggest a new mutant, S151T, which is predicted to be more selective and have higher affinity for the pre-miR-21 sequence than the S151 suggested in the original design.

## Methods

### Structures building and force-field selections

We used the lowest energy structures of the NMR ensembles 2cq3 [55], 2n7x [6] and 2n82 [6] as starting structures for the simulations of the free Rbfox protein, pre-miR20b RNA and Rbfox-pre-miR20b complex, respectively.

The starting structure of Rbfox* was prepared by introducing R118D, E147R, N151S and E152T mutations into Rbfox (in both free and bound states) using the Swiss MODEL software (available at https://spdbv.vital-it.ch/) [56–59]. The starting model of pre-miR20b* was obtained by replacing the G_28_, C_30_ and G_33_ in the pre-miR20b structure with U, A and C, respectively, using the *tleap* module of Amber 16 (available as AmberTools16 at http://ambermd.org/AmberTools16-get.html) [60]. The pre-miR20b* sequence (r-G_19_GUAGUUUUU_28_GAAUC_33_ACUCUACC_41_) is equivalent to that of the pre-miR21 only in the terminal loop (nucleotides 28–33: UGAAUC). This is part of the protein-RNA interface. The remainder of the sequence does not interact with the protein and was therefore left unchanged and identical to the pre-miR20b (r-G_19_GUAGUUUUG_28_GCAUG_33_ACUCUACC_41_).

The molecules were solvated in truncated octahedral water boxes with minimal distance of 0.10 nm between solutes and the box border. The solutes were neutralized with sodium ions followed by addition of a sufficient number of Na^+^/Cl^-^ ion pairs to reach the excess salt concentration of 80 mM. Similar solvent conditions were shown to work well for other protein-RNA systems [14, 15, 20]. The topology and coordinate files for the simulations were prepared using the *tleap* module of Amber 16 [60].

TIP3P [61], Joung and Cheatham [62], and the amber ff14SB [63], and χ_OL3_ [64] force fields were used for water, ions, proteins, and RNA respectively. This combination has shown satisfactory behavior with other protein-RNA complexes [14].

We performed also a second set of MD simulations of the free pre-miR20b RNA. These were carried out in exactly the same way as the first set except that they included the recently suggested modification of van der Waals oxygen radii for organic phosphates (atom types O2, OH, and OS)[21], along with the OPC water model [22].

### MD simulations protocol

All systems were subjected to energy minimization, and equilibration using a standard equilibration protocol [20]. In order to reduce the likelihood of instabilities in the production runs [14], NMR restraints, when available, were applied in the early stages of the majority of the simulations of the pre-miR20b RNA (Table 1, sim. 3–7) and of the Rbfox•pre-miR20b complex simulations (Table 1, sim. 9–13). Specifically, after the initial standard equilibration, the systems were simulated in the following way: 0–100 ns—all available NMR hydrogen restraints (both inter- and intra-molecular NOE interactions) were utilized, 100–120 ns—only protein–RNA (intermolecular NOE) restraints were utilized, and after 120 ns—entirely unrestrained simulations were conducted. The aim of the procedure is to guarantee a sufficient equilibration of the systems before data is gathered. Since the restraints are lifted in the later stages of the simulations, they do not bias the results. Only the primary NMR data (NOE distance restraints) were used, and were introduced with a flat-well potential [14]. Earlier, this approach was shown to be able to prevent the abrupt structural disruptions which can otherwise occur in beginning of MD simulations of protein-RNA complexes. By giving the structures more time to relax without immediate deviations from the NMR ensemble, it is possible to achieve more stable simulations of protein-RNA complexes [14]. Some simulations were also performed without the initial use of NMR restraints (Table 1, sim. 2 and 8). For detailed discussion of this protocol see [14].

Covalent bonds involving hydrogen atoms were constrained using the SHAKE algorithm [65]. Periodic boundary conditions and a 2 fs integration step were employed. The particle mesh Ewald (PME) approach[66] was used for handling electrostatic interactions. The cut-off distance of the non-bonded Lennard-Jones interactions was 0.9 nm. We used the Nose−Hoover thermostat [67] and Andersen−Parrinello−Rahman barostat [68] to maintain the systems at a temperature of 298 K and pressure of 1 bar, respectively. The completely unrestrained simulations were performed using GROMACS 5.1 (http://www.gromacs.org) [69]. Simulation runs initially using the NMR restraints were performed with the pmemd module of AMBER 14 (http://ambermd.org) [70].

### Replica Exchange simulations of the Rbfox*•pre-miR20b* complex

In order to provide proper sampling of the Rbfox*•pre-miR20b* binding interface conformational space, we performed two distinct Replica Exchange with Solute Scaling (REST2)[17] simulations. The method is based on a modification of the potential energy, so that the interactions between solute atoms are scaled by a factor λ, solvent–solvent interactions remain unscaled, and solute–solvent interactions are scaled by λ^1/2^. Scaling the energy by a factor λ is equivalent to scaling of the temperature by 1/λ. Thus, in the case of REST2, only the solute atoms are effectively heated up in REST2. Solvent–solvent interactions that typically contribute the most to the energy differences between replicas, do not contribute to exchanges, allowing to effectively reduce the number of replicas and the computational cost [17].

In a first simulation run (Table 1, sim. 15 (REST2 PS)), we explored the possibility to enhance sampling of the mutated binding interface only, by rescaling the force field parameters of the nucleotides A_30_ and G_33_ along with their flanking phosphates and the protein residues within 0.5 nm of those nucleotides (the complete list of included atoms is reported in S1 Table). Eight replicas were simulated with scaling factor λ ranging from 1 (reference replica) to 0.6, according to a geometric distribution, and leading to an average acceptance rate of 22%. Each replica was simulated for 2 μs, giving a cumulative time of 8 x 2 μs = 16 μs. For this simulation, an in-house modified Amber 16 version was used and the same simulation setup described above was adopted.

The above-proposed simulation protocol requires decoupling the degrees of freedom of the binding interface from rest of the system, but this procedure might affect fundamental molecular properties such as electrostatics and hydrophobicity [71]. Therefore, to test the accuracy of the calculations, a second REST2 simulation was conducted using a standard protocol, namely rescaling the force-field parameters of the entire solute. In this case, the Hamiltonian Replica Exchange (H-REX) code [71] as implemented in the Plumed-HREX patch of Gromacs 5.1 (https://plumed.github.io/doc-v2.3/user-doc/html/hrex.html)[71] was used. Sixteen replicas of the system were simulated, with the setup described above. A geometrical distribution of sixteen λ values ranging from 1 to 0.7 was used, which resulted in an average acceptance rate of ~20%. Each replica was simulated for 1 μs (Table 1, sim. 16 (REST 2)).

A cluster analysis was performed to identify the most populated conformers in the total simulated ensemble. In order to ensure that the clusters found would be consistent across both REST2 runs, clustering was performed on the combined trajectory obtained from the two reference (unbiased) trajectories. The *k-means* clustering algorithm implemented in cpptraj module [72] of Amber 16 [60] was used based on the Root Mean Square Deviation (RMSD) of the interface of the protein-RNA complex (nucleotides 28–33 and the amino-acids residues within 0.45 nm from those nucleotides). The combined clustering results were also parsed to obtain results for each individual simulation [73, 74].

A representative structure for each cluster was identified as the centroid conformer of the cluster (i.e., the trajectory frame with the lowest cumulative RMSD distance to every other point in the cluster). Subsequently, two additional unbiased MD simulations (Table 1, sim. 17–18) were started from the representative structures of the two most populated clusters (accounting for ~44% of all structures). Here we used Gromacs 5.1 [69] and the same protocol described above.

To compare the conformational space sampled by the two REST2 simulations and their efficiency with respect to conventional MD, we estimated the probability density *ρ(x)* of observing the system in a state *x* using a Gaussian kernel density estimate [75] (Gaussian KDE) along two collective variables (CV) [76].

Overall changes are described by the differences in the distribution of reciprocal interatomic distances (DRID)[77] with respect to the representative structure of the most populated cluster. The distribution is evaluated from the inverse intra-molecular distances between all the C_a_ and the P atoms of the protein and RNA. For each C_a_ and P inverse distance distribution, three features are extracted: the mean, the square root of the central moment, and the cube root of the third central moment. This assigns a feature matrix $v_{n}\in R^{3\times N}$ to each configuration *n*. The difference between configuration *n* and the reference structure is then

$$DRID=\frac{1}{3N}\sum_{i=1}^{N}\left\|v_{n}^{\left(∙,i\right)}-v_{0}^{\left(∙,i\right)}\right\|$$ (1)

where N is the number of residues, $v_{n}^{\left(∙,i\right)}$ denotes the feature vector of the *i*th C_a_ or P atom in ***v***_*n*_, and ***v***_0_ is the feature matrix of the reference configuration.

Local changes were captured from the fraction of conserved contacts Q between the protein and the RNA at the binding interface. *Q* is defined via a list of contact pairs between the heavy atoms *i* of residues 28–33 of the RNA loop and the heavy atoms *j* of the protein residues:

$$Q(x)=\frac{1}{N}\sum_{\left(i,j\right)}\frac{1}{1+exp[\beta (r_{ij}-\lambda r_{ij}^{0})]}$$ (2)

where the sum runs over N pairs of contacts (*i*,*j*), *r*_*ij*_(*x*) is the distance between *i* and *j* in configuration *x*, $r_{ij}^{0}$ is the distance between *i* and *j* in the reference conformation, *β* is a smoothing parameter taken to be 0.5 nm and the factor *λ*, taken to be 1.8 as default [78], accounts for fluctuations when the contact is formed.

The DRID feature vector and the fraction of native contact were obtained using the MDtraj code (http://mdtraj.org/) [79].

### MD and Free energy simulations of the S151T Rbfox*•pre-miR20b* complex

A model of the S151T Rbfox*•pre-miR20b* complex was prepared from the representative structure of the most populated cluster of the Rbfox*•pre-miR20b* complex simulations (see above for details). A threonine residue at position 151 was introduced using the Swiss MODEL software [56–59] and two standard independent MD simulations (Table 1, sim. 19–20) were conducted using the same protocol as described above.

The free energy difference associated with the S151T mutation (ΔΔG) was computed according to the thermodynamics cycle equation: ΔΔG = ΔG_co_−ΔG_s_ = ΔG^S151^–ΔG^S151T^. The ΔG_co_ and ΔG_s_ represent the results of the non-equilibrium alchemical calculations[52] of the S151T protein-RNA complex and of the free protein state, respectively. The ΔG^S151^ and ΔG^S151T^ are the dissociation energy of the Rbfox*•pre-miR20b* and of the S151T Rbfox*•pre-miR20b* complex, respectively.

The free energy calculations were conducted in a following fashion: From the equilibrium production simulations of the Rbfox*•pre-miR20b* complex (Table 1, sim. 17) and of the Rbfox* protein (Table 1, sim. 14), 10 conformations were extracted equidistantly in time, and, for every configuration, a hybrid structure/topology for the S151T mutation was generated using the pmx utilities (http://pmx.mpibpc.mpg.de/) [51, 80]. Subsequently, a 1 ns MD simulation for every configuration was performed to equilibrate the velocities on the introduced dummy atoms.

From each equilibrium simulation, 100 snapshots were extracted equidistantly in time, and finally, a 200 ps (Table 1, sim. 21 (FE)) or 1 ns (Table 1, sim. 22 (FE)) alchemical transition was initiated to morph the system from one physical state to the other. The alchemical transformations were performed in both directions: S151 to S151T and vice versa. A soft-core function with the default parameters (α = 0.3, σ = 0.25, *p* = 1)[51, 81] was used for the non-bonded interactions during the non-equilibrium transitions. The work values from the non-equilibrium transitions were used to calculate free energy differences based on the Crooks theorem [82] utilizing the maximum likelihood estimator [83]. The protocol described above was applied to all the alchemical simulations.

Table 1 reports the complete list of the simulations performed in this work (overall more than 50 μs of molecular simulation).

### Simulation analysis

Hydrogen bonds were analyzed using the cpptraj [72] module of AMBER 16 (available as AmberTools16 at http://ambermd.org/AmberTools16-get.html) [60]. We used a distance cut-off of 0.35 nm between the relevant heavy atoms and an angle cut-off of 135° for the intervening hydrogen atom. These interactions are characterized by the percentage of the trajectory during which they are observed (i.e. occupancy). Aromatic amino acids and nucleobases were considered to form stacking interactions if the distance between their centers of mass was less than 0.5 nm and the angle between the two planes was less than 30°.

RNAs base pair, base-pair steps and the ion distributions around the RNA helical axes in the simulated systems were analyzed with the Curves+ program [31] and the Canal and Canion utilities (available at https://bisi.ibcp.fr/tools/curves_plus/). Average ion molarities were calculated by setting the groove limit at a radius of 0.11 nm from the RNA helical axis, while the angular limits were determined by the average position of the sugar C1’ atoms.

Deviations relative to the initial RNA structure were calculated using the *ε*RMSD metric [19], a recently suggested RNA-specific structural metrics that is considered more robust than the notoriously insensitive and ambiguous RMSD [84, 85]. Two structures with *ε*RMSD of 0.7 or lower are considered to be significantly similar [19].The εRMSD was calculated using the baRNAba package (available at https://github.com/srnas/barnaba).

The protein’s deviations from the initial structure were analyzed in terms of the RMSD, calculated using the cpptraj [72] module of Amber 16 [60]. The protein backbone conformational plasticity was calculated in terms of PAD_ω_ angle from the T-PAD analysis (freely available upon request) [18]. The latter provides a quantitative analysis of local plasticity of individual residues in terms of the angular dispersion ω, which is the sum of the Ramachandran angles Φ and ψ. Moreover, it allows distinction between backbone local fluctuations and conformational transitions (from one region of the Ramachandran plot to another) even when they occur with the same amplitude [18]: the tag “F” is assigned to fluctuations, “T” to long transitions (i.e., contributing more than 30% of the simulation time) and “t” to short transitions (i.e., contributing less than 30% of the simulation time). This analysis has been successfully used in the past to evaluate proteins backbone fluctuations from MD simulation trajectories and NMR structures [86].

The conformational entropy has been estimated by calculating the chain’s conformational entropy from the distribution of the backbone (φ, ψ) and side-chains rotameric angles, [χ_n_] following ref. [87]. The calculation has been performed on the trajectories of Rbfox and Rbfox* free and in complex with RNA.

### NMR observables

Protein‘ chemical shifts (CS) were predicted using SHIFTX2 v. 1.07 (http://www.shiftx2.ca/) [37, 38]. LARMOR^D^ software (https://brooks.chem.lsa.umich.edu/) [36] was used for RNA. In the SHIFTX2 program the sequence information is not used in the prediction, so that the predictions are identical to those of the SHIFTX+ program (http://www.shiftx2.ca/performance.html). We run SHIFTX2 and LARMOR^D^ on each frame extracted from the un-restrained simulations every 10 ps of the free pre-miR20b and of the Rbfox•pre-miR20b complex, for which experimental CS are available. The chemical shifts predictions for these 48,000 pre-miR20b and 48,000 Rbfox•pre-miR20b conformers were then linearly averaged to make a final prediction for the protein’ ^13^C_a_, ^13^C’, ^13^C_b_, ^15^N and for the RNA’s ^13^C and ^1^H CS.

For the set of experimental upper bound distance restraint *r*_NOE_, the simulated NOE’s 〈*r*_*i*,*j*_〉 were calculated according to:

$$\langle r_{i,j}\rangle ={\left(\frac{1}{N_{f}}\sum {\left(r_{i,j}\right)}^{-6}\right)}^{-\frac{1}{6}}$$ (3)

where *r*_*i*,*j*_ is the interatomic distance between atoms *i* and *i*, and the sum runs over the *N*_*f*_ trajectories frames. The average distance violation was defined as:

$$\frac{1}{N_{\mathrm{NOE}}}\sum \left(r_{\mathrm{NOE}}-\langle r_{i,j}\rangle \right)\;\mathrm{if}\;r_{NOE}<\langle r_{i,j}\rangle$$ (4)

where the sum runs over all reported intermolecular NOE-based distance restraints. The conformers with best match with the NOEs upper bounds were then selected to produce an “MD-adapted structure ensemble” using the same protocol as in [14]. In particular, we used the combined simulation trajectories of the Rbfox•pre-miR20b complex and from each we selected 10% of frames with fewest NOE violations. *K-means* clustering algorithm was used to cluster this group of frames based on the RMSD of the complex. The representative structures of the 20 clusters obtained constitute the “MD-adapted structure ensemble”: sets of atomic coordinates (deposited as PDB files at https://doi.org/10.5281/zenodo.1297931) that capture the flexibility and the conformers suggested by MD simulations while still retaining the highest possible level of agreement with the primary NMR data.

## Supporting information

S1 Text Are similar types of motion sampled similarly across different MD simulations?. (PDF)

S2 Text Convergence of REST2 and REST2 PS simulations. (PDF)

S1 Fig Overlap of principle components (PCs) for independent simulations. Histograms from PCs analysis in Cartesian space calculated from the trajectories with independent projection of the PCs on the separate trajectories of the pre-miR20b (A, Table 1, sim. 2–6) and (B) of the Rbfox•pre-miR20b (Table 1, sim. 9–13).
(PDF)

S2 Fig Root mean square deviation (RMSD). calculated over heavy atoms with respect to the initial structure of the Rbfox protein in simulations of (A) the free state (Table 1, sim.1) and (B) bound to pre-miR20b RNA (Table 1, sim. 2–7).
(PDF)

S3 Fig Entropy differences between Rbfox* and Rbfox, free (F) and in complex with RNA (C). Details of the calculations are reported in the Materials and Methods section.
(PDF)

S4 Fig (A) RNA backbone dihedral angles calculated over the aggregated simulations of the pre-miR20b Rbfox complex (Table 1, sim. 8–13). The green dots indicate the values of the angles in the lowest energy structure of the NMR ensemble of the Rbfox•pre-miR20b complex from which the simulations where started. (B) εRMSD of the pre-miR20b loop sequences U_27_GGCAUG_33_ (left) and G_28_GCAUG_33_ (right) in complex with Rbfox versus time in the six MD simulations performed (Table 1, sim. 8–13).
(PDF)

S5 Fig Base pair (bp) and base pair steps (bps) of pre-miR20b in complex with Rbfox. (A) Bp and (B) bps parameters for base pairs G_20_-C_40_, U_21_-A_39_, A_22_-U_38_, G_23_-C_37_, calculated over the aggregated simulations (Table 1, sim. 8–13; dark blue) and NMR ensemble (light blue).
(PDF)

S6 Fig (A) 2D representation of the free pre-miR20b loop structure. (B) Time development of the *ε*RMSD of the pre-miR20b loop (r-U_27_GGCAUG_33_) in the three χ_OL3_ -CP-OPC MD simulations (Table 1, sim. 1–3 (χ_OL3_ -CP-OPC)). C. RNA backbone dihedral angle histograms calculated over the aggregated simulations. The green dots indicate the values of the angles in the lowest energy structure of the NMR ensemble 2n7x from which the simulations were started.
(PDF)

S7 Fig Conformations of the pre-miR20b loop in simulations 2–7 (as listed in Table 1). The loop conformation in the initial structure is shown as the grey overlay.
(PDF)

S8 Fig Base pair (bp) and base pair steps (bps) of pre-miR20b RNA in the free state. (A) bp and (B) bps parameters for base pairs G_20_-C_40_, U_21_-A_39_, A_22_-U_38_, G_23_-C_37_, U_24_-U_36_, U_25_-C_35_, U_26_-A_34_, calculated over the entire MD (dark blue) and NMR (light blue) ensembles.
(PDF)

S9 Fig **Average Na**^**+**^ **distribution** for the (A) free pre-miR20b (Table 1, simulations 2–7) and (B) its complex with the Rbfox protein (Table 1, simulations 8–13), as a function of the distance from the helical axis (R). The results are plotted as molarities as shown by the color bars, with blue to yellow scale indicating increasing values. The vertical white line indicates the radial position of the phosphorus atoms.
(PDF)

S10 Fig Stacking interactions in the Rbfox•pre-miR20b complex. Stacking geometries are described by the center of mass distance d and the angle θ between the planes of the bases and amino acid side chain. Amino acid and nucleobase are considered stacked if d*<* 0.5 nm and θ <30°. The distributions are calculated for G_29_-R184, G_29_-F126, U_32_-H120 and G_33_-F160 pairs in the individual unrestrained trajectories (Table 1, sim. 8–13).
(PDF)

S11 Fig Calculated and experimentally measured [6] chemical shifts (CS) for pre-miR20b in the free state (A), for pre-miR20b in complex with the Rbfox (B) and for the Rbfox protein (C). The CS have been calculated using the SHIFTX+ [88] for the protein and LARMOR^D^ [36] for the RNA. Additional details are reported in the Materials and Methods section.
(PDF)

S12 Fig Comparison between calculated and experimental [6] ^13^C’, ^13^Ca, ^13^Cb and ^15^N CS of Rbfox in the free state and bound to pre-miR20b. The grey square represents the standard deviation. Additional details on the calculations are reported in the Materials and Methods section.
(PDF)

S13 Fig Distributions of CS predicted by SHIFTX+ [88] for F150 and F158 ^15^N from the MD simulations of the Rbfox in complex with pre-miR20b (see Materials and Methods for details) and values of the χ_1_ angles of the same residues in trajectory 9. The blue dot represents the calculated average value; the red one corresponds to the experimental value. A similar behaviour is observed in the other simulations performed on the system.
(PDF)

S14 Fig Comparison of calculated and experimental chemical shifts for the (A) ^13^C and (B) ^1^H atoms of pre-miR20b in the free state (Table 1, sim. 2–7). Representation as in S12 Fig.
(PDF)

S15 Fig Comparison of calculated and experimental chemical shifts for the ^13^C and ^1^H atoms of pre-miR20b bound to Rbfox (Table 1, sim. 8–13). Representation as in S12 Fig.
(PDF)

S16 Fig Stacking interactions in the Rbfox*•pre-miR20b* complex. The distributions are calculated for G_29_-R184, G_29_-F126, U_32_-H120 and C_33_-F160 pairs in replica exchange (Table 1, sims. 15–16) and unbiased MD (Table 1, sims, 17–18) simulations.
(PDF)

S17 Fig Probability density of sampling the conformational landscape of the Rbfox*•pre-miR20b* complex in the two-dimensional space of Native Contacts and of DRID (see Method sections for details) for plain MD, conventional (standard) REST2 and REST2 with partial scaling (REST2 PS), respectively. (PDF)

S18 Fig Rbfox•pre-miR20b* complex. (A) Top view of the structure in the simulation (Table 1, sim. 23). (B) Details of the stacking interactions of G_29_ with F126 and R184.
(PDF)

S19 Fig Rbfox*•pre-miR20b complex. (A) Top view of the structure in the simulation (Table 1, sim. 24). (B) Details of the H-bond interactions (dashed blue lines) involving G_33_, R147 and D118.
(PDF)

S1 Table Selected region for the “Partial Scaling” REST2 simulation of the Rbfox*•pre-miR20b* complex. (PDF)

S2 Table NOE-upper bounds violations (*v*) for pre-miR20b in the free state (Table 1, sim. 2–7), Rbfox (Table 1, sim. 1) and Rbfox•pre-miR20b complex (Table 1, sim. 8–13) calculated along all individual MD trajectories and on the “full ensemble” obtained by merging all the unrestrained parts of the initially restrained trajectories. (PDF)
