# Supplementary material for: Molecular basis for the increased affinity of an RNA recognition motif with re-engineered specificity: A molecular dynamics and enhanced sampling simulations study
Source: PLoS Comput Biol. 2018 Dec 6;14(12):e1006642. doi: 10.1371/journal.pcbi.1006642 (PMC6307825; doi:10.1371/journal.pcbi.1006642)
Supplement: S1 Text — (PDF) [file pcbi.1006642.s001.pdf]

Supporting Information

Molecular basis for the increased affinity of an RNA recognition motif with re-engineered specificity: A molecular dynamics and enhanced sampling simulations study.

Anna Bochicchio*[¶1,#a], Miroslav Krepl*[¶3], Fan Yang[5,#b], Gabriele Varani[5], Jiri Sponer[3,4], Paolo Carloni*[1,2].

[1]Computational Biomedicine, Institute for Advanced Simulation IAS-5 and Institute of Neuroscience and Medicine INM-9, Forschungszentrum Jülich, 52425 Jülich, Germany

[2]JARA-HPC, Jülich Supercomputing Centre, Forschungszentrum Jülich GmbH, 52425 Jülich, Germany

[3]Institute of Biophysics of the Czech Academy of Sciences, Kralovopolska 135, 612 65 Brno, Czech Republic

[4]Regional Centre of Advanced Technologies and Materials, Department of Physical Chemistry, Faculty of Science, Palacky University Olomouc, 17. Listopadu 12, 771 46 Olomouc, Czech Republic

[5]Department of Chemistry, University of Washington, Seattle, 36 Bagley Hall Seattle, 98195-1700 Washington, United States of America

[#a]Current Address: Computational Biology, Department of Biology, Friedrich-Alexander University Erlangen-Nürnberg, 91058 Erlangen, Germany

[#b]Current Address: School of Life Science and Technology, Harbin University of Technology, 92 West Dazhi Street , Nan Gang District, Harbin 150080, China

[*]Corresponding author
E-mail: p.carloni@fz-juelich.de (PC)
E-mail: anna.bochicchio@fau.de (AB)
E-mail: krepl@seznam.cz (MK)

[¶]These authors contributed equally to this work

S1 Text. Are similar types of motion sampled similarly across different MD simulations? Principal component (PC) analysis can reveal the dominant modes of motion in a simulation, by identifying eigenvectors of the so-called covariance matrix [1, 2]. Comparing PC projections provide a metric of similarity between the dominant modes of motion across independent MD simulations [1, 2]. This has been done here, using the cpptraj module[3] of Amber 16 [4], to assess whether the same types of motion are sampled similarly across individual MD trajectories of the pre-miR20b and Rbfox•pre-miR20b systems. First, to ensure that the eigenvectors obtained from each simulation match, we calculated the covariance matrix for non-hydrogen atoms using all the trajectories combined. Each frame was RMS-fit to the overall average coordinates in order to remove global rotational and translational motions. Next, the projection along the eigenvectors of each coordinate frame from the first simulation trajectory was calculated; this was then repeated for the second simulation trajectory and so on. Finally, at each frame t, a histogram for each simulation of the PC projection values for a given PC is constructed. Figure S1 shows the overlap of histograms of the PC projections for the three most dominant modes of motion. Since the histograms are very similar in all the cases, these results suggest that the same types of motion are sampled in all independent simulations.

References

1. Bergonzo C, Henriksen NM, Roe DR, Swails JM, Roitberg AE, Cheatham TE. Multidimensional Replica Exchange Molecular Dynamics Yields a Converged Ensemble of an RNA Tetranucleotide. Journal of Chemical Theory and Computation. 2014;10:492-9.
2. Roe DR, Bergonzo C, Cheatham TE. Evaluation of Enhanced Sampling Provided by Accelerated Molecular Dynamics with Hamiltonian Replica Exchange Methods. The Journal of Physical Chemistry B. 2014;118(13):3543-52.
3. Roe DR, Cheatham TE. PTRAJ and CPPTRAJ: Software for Processing and Analysis of Molecular Dynamics Trajectory Data. Journal of Chemical Theory and Computation. 2013;9:3084-95.
4. Case DA, Botello-Smith W, Cerutti DS, Cheatham TE III, Darden TA, Duke RE, Giese TJ, Gohlke H, Goetz AW,Homeyer N, Izadi S , Janowski P, Kaus J, Kovalenko A, Lee TS, LeGrand S, Li P, Lin C, Luchko T, Luo R, Madej B, Mermelstein D, Merz KM, Monard G, Nguyen H, Nguyen HT, Omelyan I, Onufriev A, Roe DR, Roitberg A, Sagui C, Simmerling CL, Swails J, Walker RC, Wang J, Wolf RM, Wu X, Xiao L, York DM, Kollman PA. AMBER 2016. San Francisco: University of California; 2016.
