# Supplementary material for: Molecular basis for the increased affinity of an RNA recognition motif with re-engineered specificity: A molecular dynamics and enhanced sampling simulations study
Source: PLoS Comput Biol. 2018 Dec 6;14(12):e1006642. doi: 10.1371/journal.pcbi.1006642 (PMC6307825; doi:10.1371/journal.pcbi.1006642)
Supplement: S2 Text — (PDF) [file pcbi.1006642.s002.pdf]

S17 Text. Convergence of REST2 and REST2 PS simulations. The most widely sample conformations were identified by a cluster analysis on the combined reference trajectories from the two simulations. The clustering results were then partitioned based on the single trajectories to compare populations of structures in the two simulation to determine convergence. The cluster populations between the two trajectories agree well (with a maximum difference in fraction population of about 9%) for the first two clusters, which account for more than 44% of the total population. The next three most populated clusters account for 7.7, 7.0, and 3.1% of the total structures, respectively. For these more minor clusters, the agreement between the cluster populations in the two trajectories deteriorates and, in particular, clusters 5 and 6 are not found in the REST2 simulation where only the binding interface is considered for Hamiltonian scaling (REST2 PS). A direct comparison of the simulations methods' ability to explore the conformational landscape in the space of the native contacts and of the collective variable DRID (see Methods section) is shown in Figure S17. The comparison was extended to regular MD started from the representative structure of the first combined cluster. The conventional REST2 simulation samples the Rbfox*•pre-miR20b* binding interface conformations more extensively than the REST2 PS and standard MD, consistent with the results of the cluster analysis. Extending the REST2 PS simulation to 2 μs does not improve sampling. Indeed, the region explored is similar to the one visited in the first 1 μs of simulation. However, the population of the structural hydrogen bonds at the binding interface (Table 5) in the different basins of the REST2 conformational landscape (Figure S17), differs by only ~4–5%. Hence, the different basins along the Native Contacts reflect the occurrence of minor interactions at the binding interface that do not significantly alter the RNA-protein recognition mode. Nonetheless, a better evaluation of the efficiency of the methods would at the minimum require performing more independent simulations of each type and is beyond the scope of this work.
