# Supplementary material for: Molecular basis for the increased affinity of an RNA recognition motif with re-engineered specificity: A molecular dynamics and enhanced sampling simulations study
Source: PLoS Comput Biol. 2018 Dec 6;14(12):e1006642. doi: 10.1371/journal.pcbi.1006642 (PMC6307825; doi:10.1371/journal.pcbi.1006642)
Supplement: S1 Fig — Histograms from PCs analysis in Cartesian space calculated from the trajectories with independent projection of the PCs on the separate trajectories of the pre-miR20b (A, Table 1, sim. 2–6) and (B) of the Rbfox•pre-miR20b (Table 1, sim. 9–13). (PDF) [file pcbi.1006642.s003.pdf]

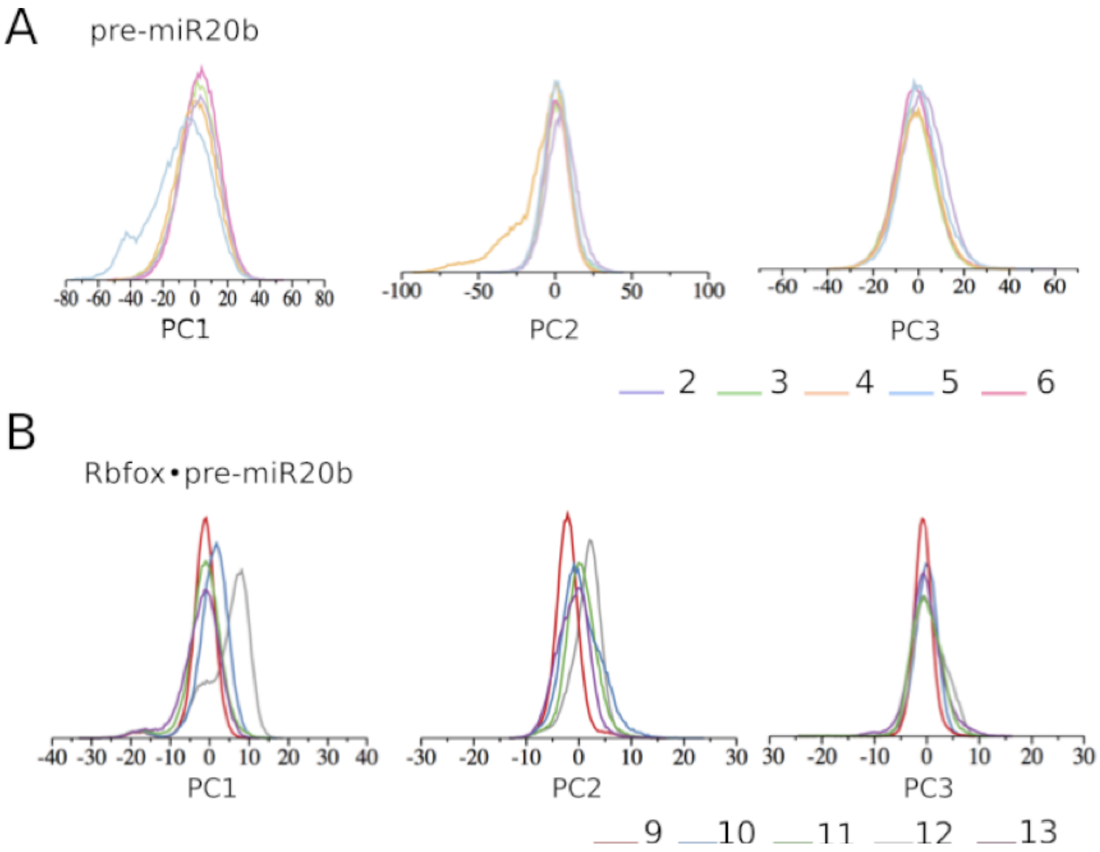

S1 Fig. Overlap of principle components (PCs) for independent simulations. Histograms from PCs analysis in Cartesian space calculated from the trajectories with independent projection of the PCs on the separate trajectories of the pre-miR20b (A, Table 1, sim. 2-6) and (B) of the Rbfox□pre-miR20b (Table 1, sim. 9-13).
