# Supplementary material for: Molecular basis for the increased affinity of an RNA recognition motif with re-engineered specificity: A molecular dynamics and enhanced sampling simulations study
Source: PLoS Comput Biol. 2018 Dec 6;14(12):e1006642. doi: 10.1371/journal.pcbi.1006642 (PMC6307825; doi:10.1371/journal.pcbi.1006642)
Supplement: S2 Fig — calculated over heavy atoms with respect to the initial structure of the Rbfox protein in simulations of (A) the free state (Table 1, sim.1) and (B) bound to pre-miR20b RNA (Table 1, sim. 2–7). (PDF) [file pcbi.1006642.s004.pdf]

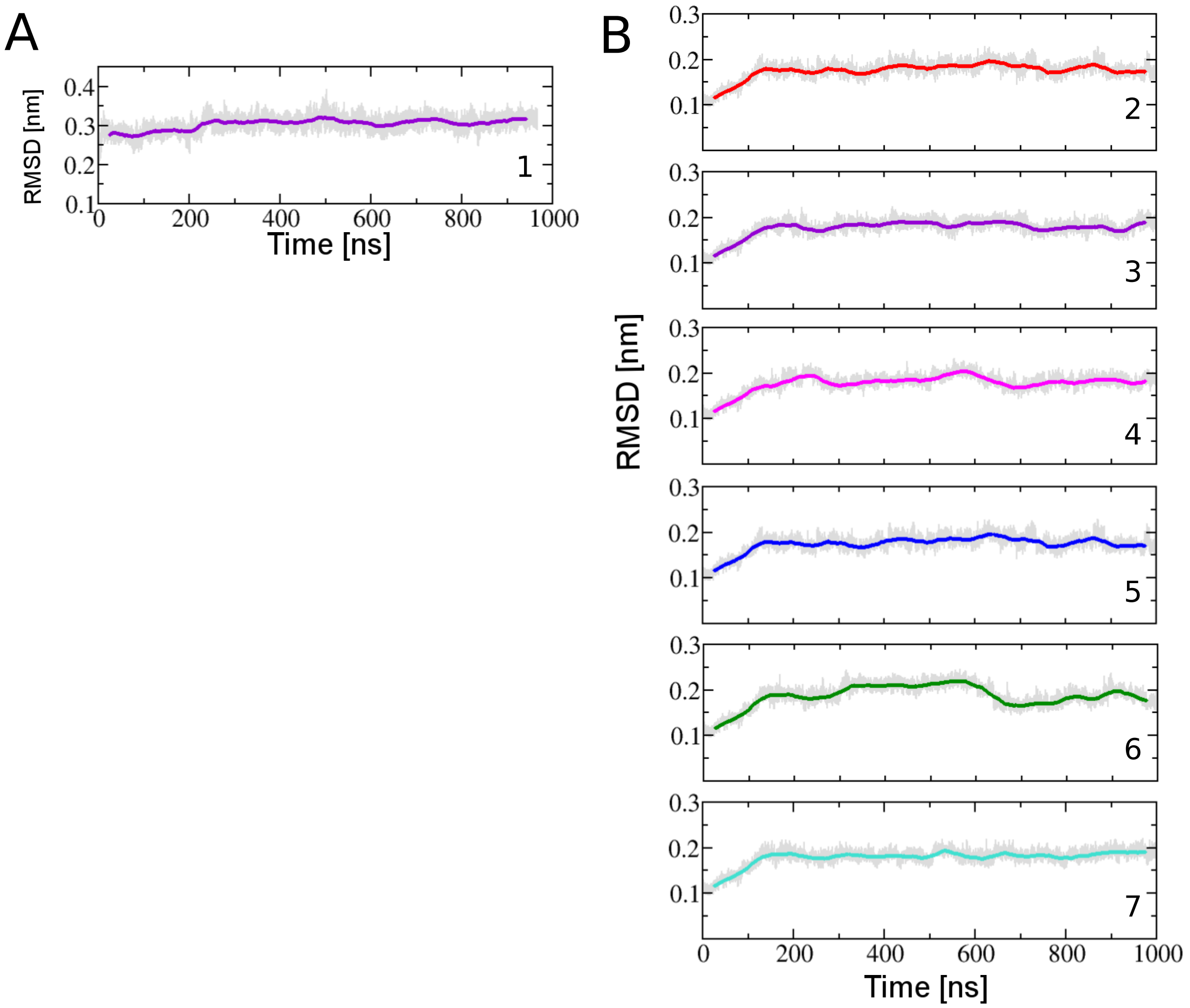

S2 Fig. Root mean square deviation (RMSD) calculated over heavy atoms with respect to the initial structure of the Rbfox protein in simulations of (A) the free state (Table 1, sim.1) and (B) bound to pre-miR20b RNA (Table 1, sim. 2-7).
