# Supplementary material for: Molecular basis for the increased affinity of an RNA recognition motif with re-engineered specificity: A molecular dynamics and enhanced sampling simulations study
Source: PLoS Comput Biol. 2018 Dec 6;14(12):e1006642. doi: 10.1371/journal.pcbi.1006642 (PMC6307825; doi:10.1371/journal.pcbi.1006642)
Supplement: S3 Fig — Details of the calculations are reported in the Materials and Methods section. (PDF) [file pcbi.1006642.s005.pdf]

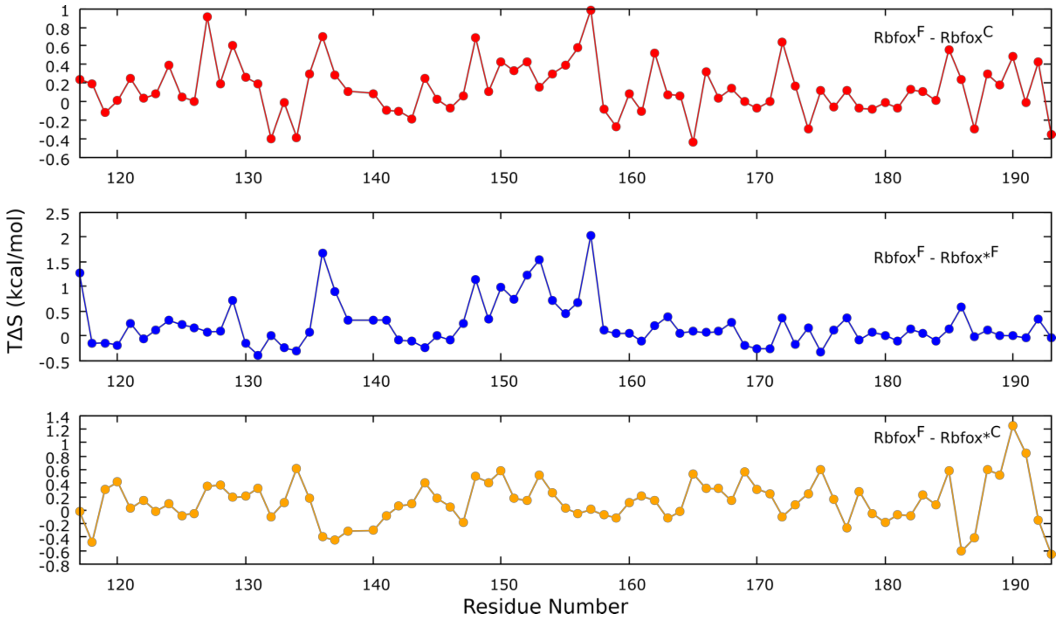

S3 Fig. Entropy differences between Rbfox* and Rbfox, free (F) and in complex with RNA (C). Details of the calculations are reported in the Materials and Methods section.
