# Supplementary material for: Molecular basis for the increased affinity of an RNA recognition motif with re-engineered specificity: A molecular dynamics and enhanced sampling simulations study
Source: PLoS Comput Biol. 2018 Dec 6;14(12):e1006642. doi: 10.1371/journal.pcbi.1006642 (PMC6307825; doi:10.1371/journal.pcbi.1006642)
Supplement: S4 Fig — (A) RNA backbone dihedral angles calculated over the aggregated simulations of the pre-miR20b Rbfox complex (Table 1, sim. 8–13). The green dots indicate the values of the angles in the lowest energy structure of the NMR ensemble of the Rbfox•pre-miR20b complex from which the simulations where started. (B) εRMSD of the pre-miR20b loop sequences U27GGCAUG33 (left) and G28GCAUG33 (right) in complex with Rbfox versus time in the six MD simulations performed (Table 1, sim. 8–13). (PDF) [file pcbi.1006642.s006.pdf]

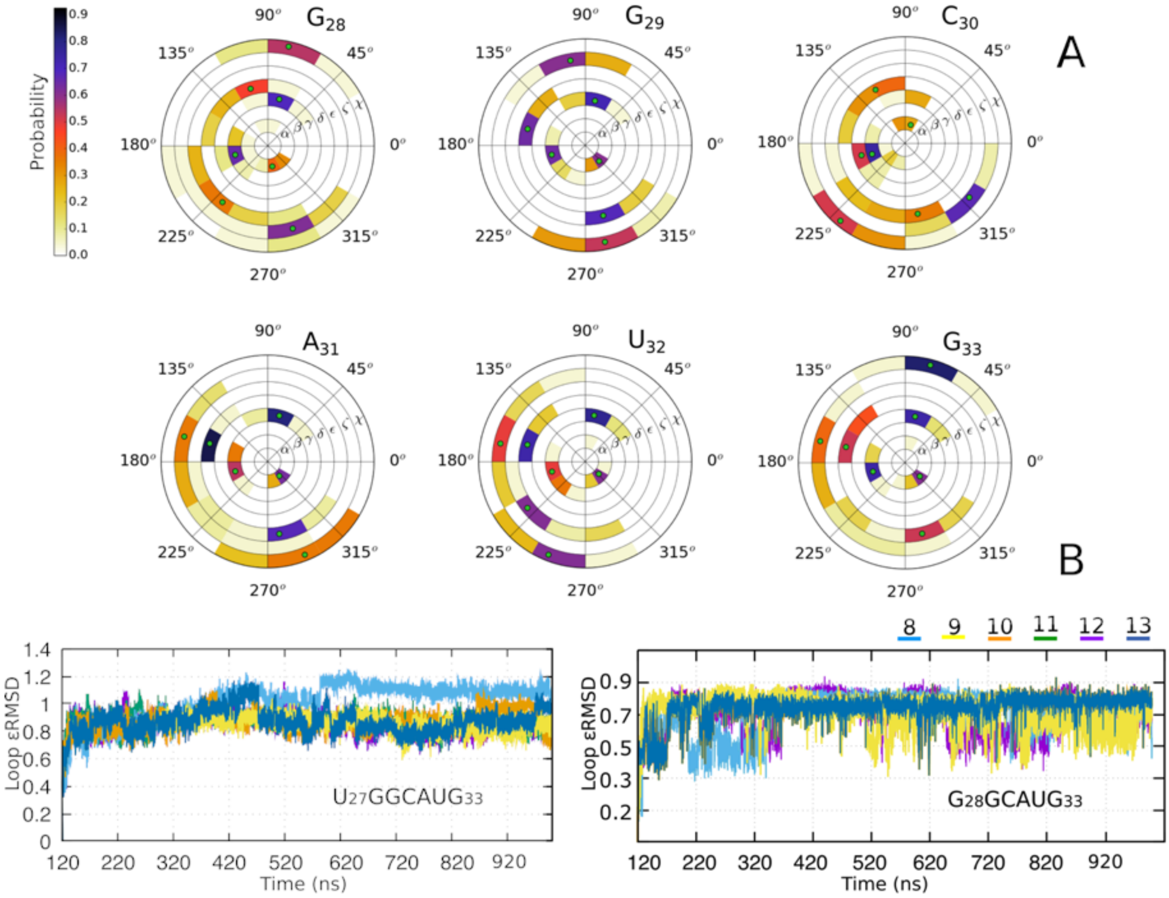

S4 Fig. (A) RNA backbone dihedral angles calculated over the aggregated simulations of the pre-miR20b Rbfox complex (Table 1, sim. 8-13). The green dots indicate the values of the angles in the lowest energy structure of the NMR ensemble of the Rbfox•pre-miR20b complex from which the simulations where started. (B) *ε*RMSD of the pre-miR20b loop sequences U$_{27}$GGCAUG$_{33}$ (left) and G$_{28}$GCAUG$_{33}$ (right) in complex with Rbfox versus time in the six MD simulations performed (Table 1, sim. 8-13).
