# Supplementary material for: Molecular basis for the increased affinity of an RNA recognition motif with re-engineered specificity: A molecular dynamics and enhanced sampling simulations study
Source: PLoS Comput Biol. 2018 Dec 6;14(12):e1006642. doi: 10.1371/journal.pcbi.1006642 (PMC6307825; doi:10.1371/journal.pcbi.1006642)
Supplement: S5 Fig — (A) Bp and (B) bps parameters for base pairs G20-C40, U21-A39, A22-U38, G23-C37, calculated over the aggregated simulations (Table 1, sim. 8–13; dark blue) and NMR ensemble (light blue). (PDF) [file pcbi.1006642.s007.pdf]

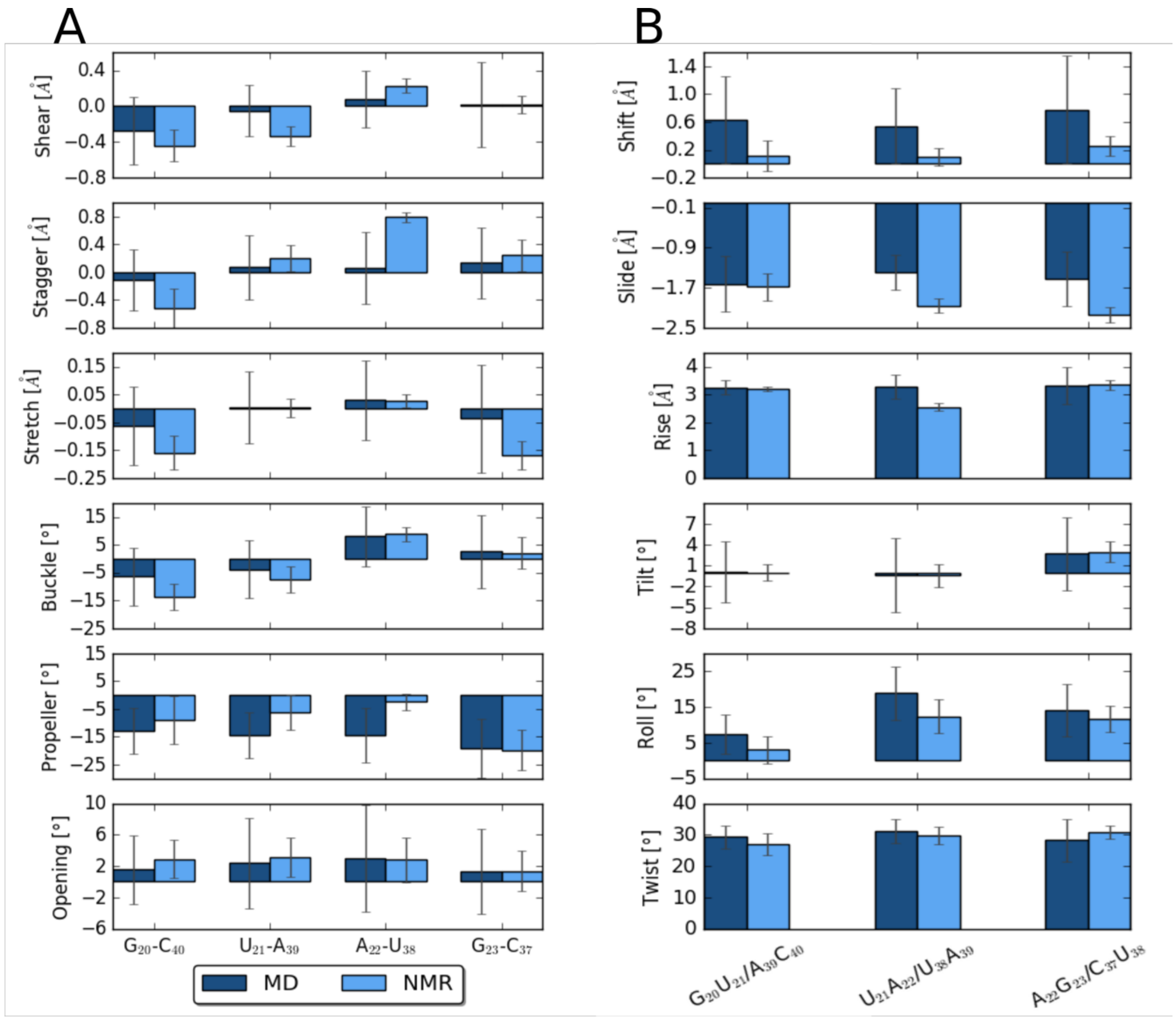

S5 Fig. Base pair (bp) and base pair steps (bps) of pre-miR20b in complex with Rbfox. (A) Bp and (B) bps parameters for base pairs G_{20}-C_{40}, U_{21}-A_{39}, A_{22}-U_{38}, G_{23}-C_{37}, calculated over the aggregated simulations (Table 1, sim. 8-13; dark blue) and NMR ensemble (light blue).
