# Supplementary material for: Molecular basis for the increased affinity of an RNA recognition motif with re-engineered specificity: A molecular dynamics and enhanced sampling simulations study
Source: PLoS Comput Biol. 2018 Dec 6;14(12):e1006642. doi: 10.1371/journal.pcbi.1006642 (PMC6307825; doi:10.1371/journal.pcbi.1006642)
Supplement: S6 Fig — (A) 2D representation of the free pre-miR20b loop structure. (B) Time development of the εRMSD of the pre-miR20b loop (r-U27GGCAUG33) in the three χOL3 -CP-OPC MD simulations (Table 1, sim. 1–3 (χOL3 -CP-OPC)). C. RNA backbone dihedral angle histograms calculated over the aggregated simulations. The green dots indicate the values of the angles in the lowest energy structure of the NMR ensemble 2n7x from which the simulations were started. (PDF) [file pcbi.1006642.s008.pdf]

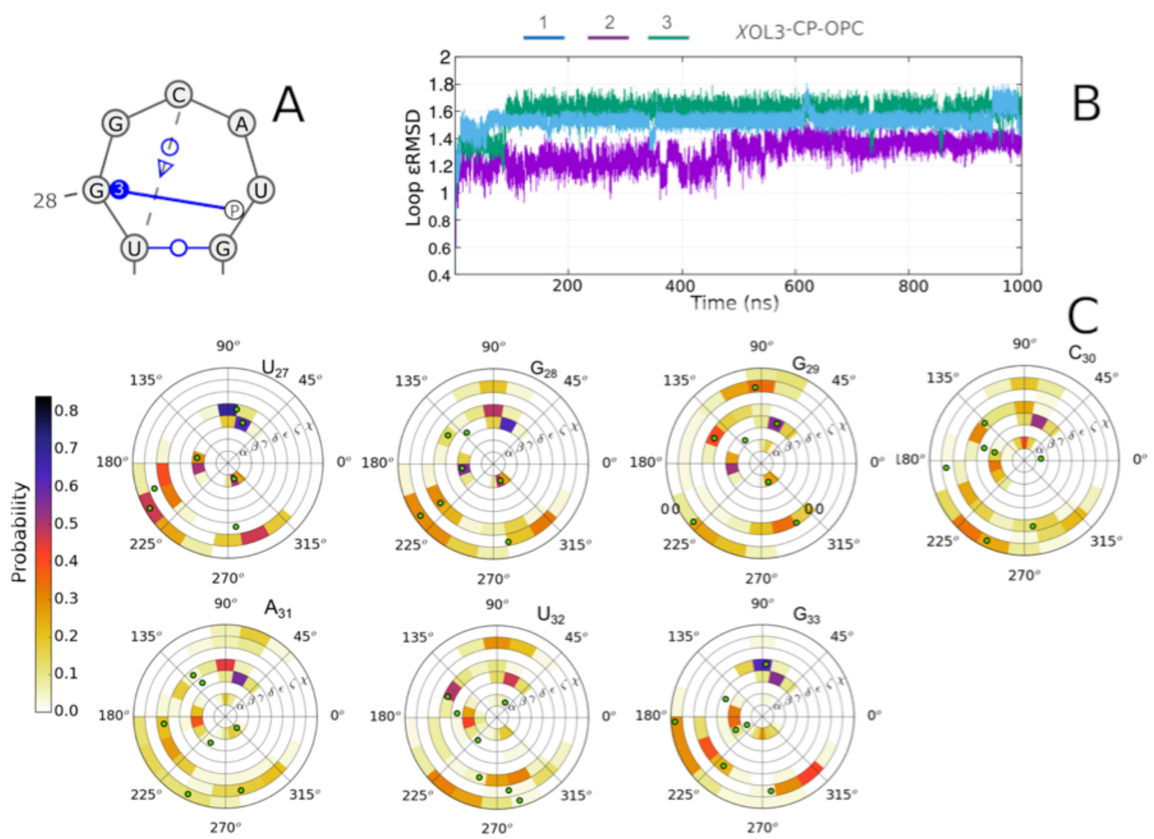

S6 Fig. (A) 2D representation of the free pre-miR20b loop structure. (B) Time development of the εRMSD of the pre-miR20b loop (r-U_{27}GGCAUG_{33}) in the three χ_{OL3}-CP-OPCMD simulations (Table 1, sim. 1-3 (χ_{OL3}-CP-OPC)). C. RNA backbone dihedral angle histograms calculated over the aggregated simulations. The green dots indicate the values of the angles in the lowest energy structure of the NMR ensemble 2n7x from which the simulations were started.
