# Supplementary material for: Molecular basis for the increased affinity of an RNA recognition motif with re-engineered specificity: A molecular dynamics and enhanced sampling simulations study
Source: PLoS Comput Biol. 2018 Dec 6;14(12):e1006642. doi: 10.1371/journal.pcbi.1006642 (PMC6307825; doi:10.1371/journal.pcbi.1006642)
Supplement: S7 Fig — The loop conformation in the initial structure is shown as the grey overlay. (PDF) [file pcbi.1006642.s009.pdf]

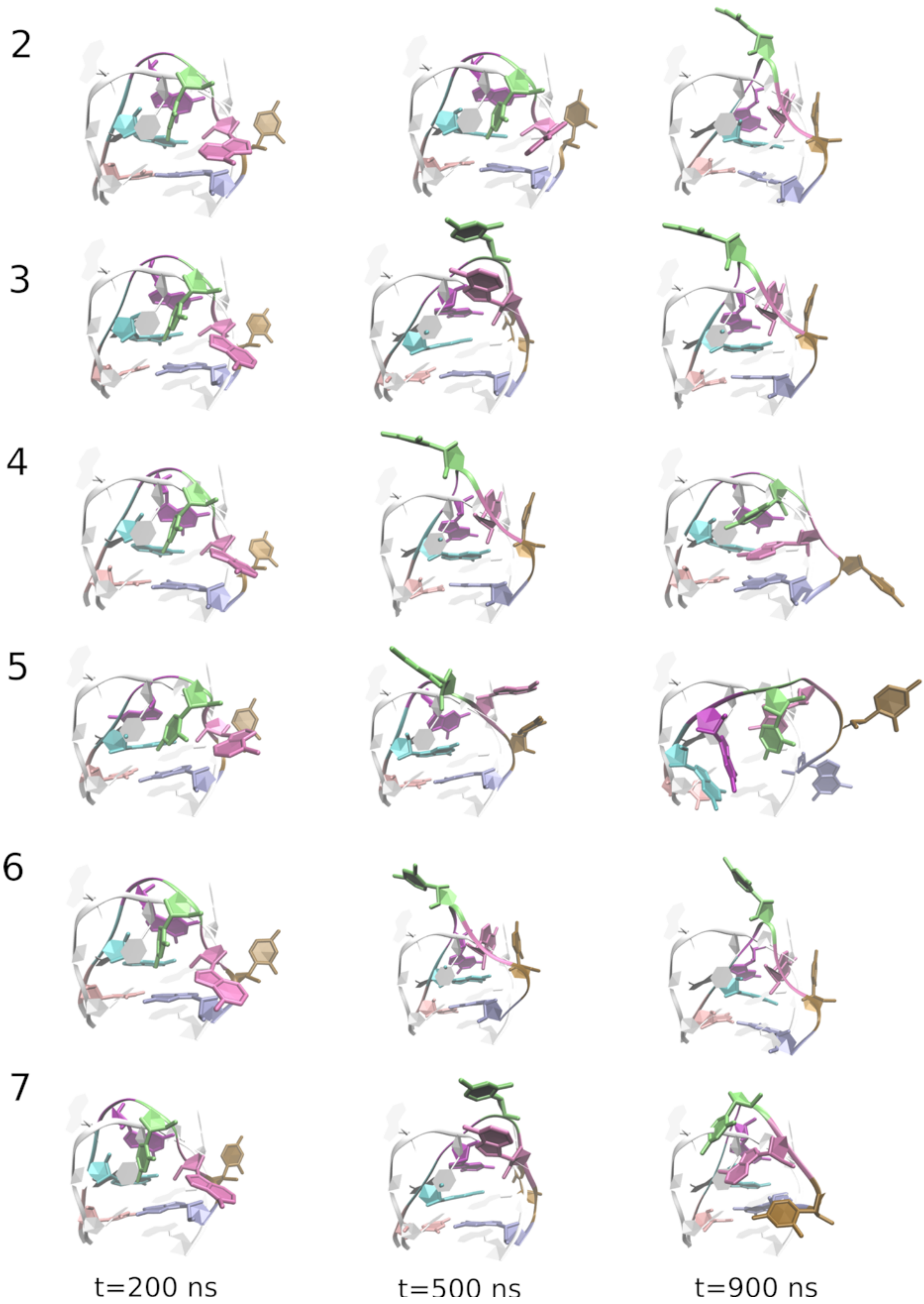

S7 Fig. Conformations of the pre-miR20b loop in simulations 2-7 (as listed in Table 1). The loop conformation in the initial structure is shown as the grey overlay.
