# Supplementary material for: Molecular basis for the increased affinity of an RNA recognition motif with re-engineered specificity: A molecular dynamics and enhanced sampling simulations study
Source: PLoS Comput Biol. 2018 Dec 6;14(12):e1006642. doi: 10.1371/journal.pcbi.1006642 (PMC6307825; doi:10.1371/journal.pcbi.1006642)
Supplement: S8 Fig — (A) bp and (B) bps parameters for base pairs G20-C40, U21-A39, A22-U38, G23-C37, U24-U36, U25-C35, U26-A34, calculated over the entire MD (dark blue) and NMR (light blue) ensembles. (PDF) [file pcbi.1006642.s010.pdf]

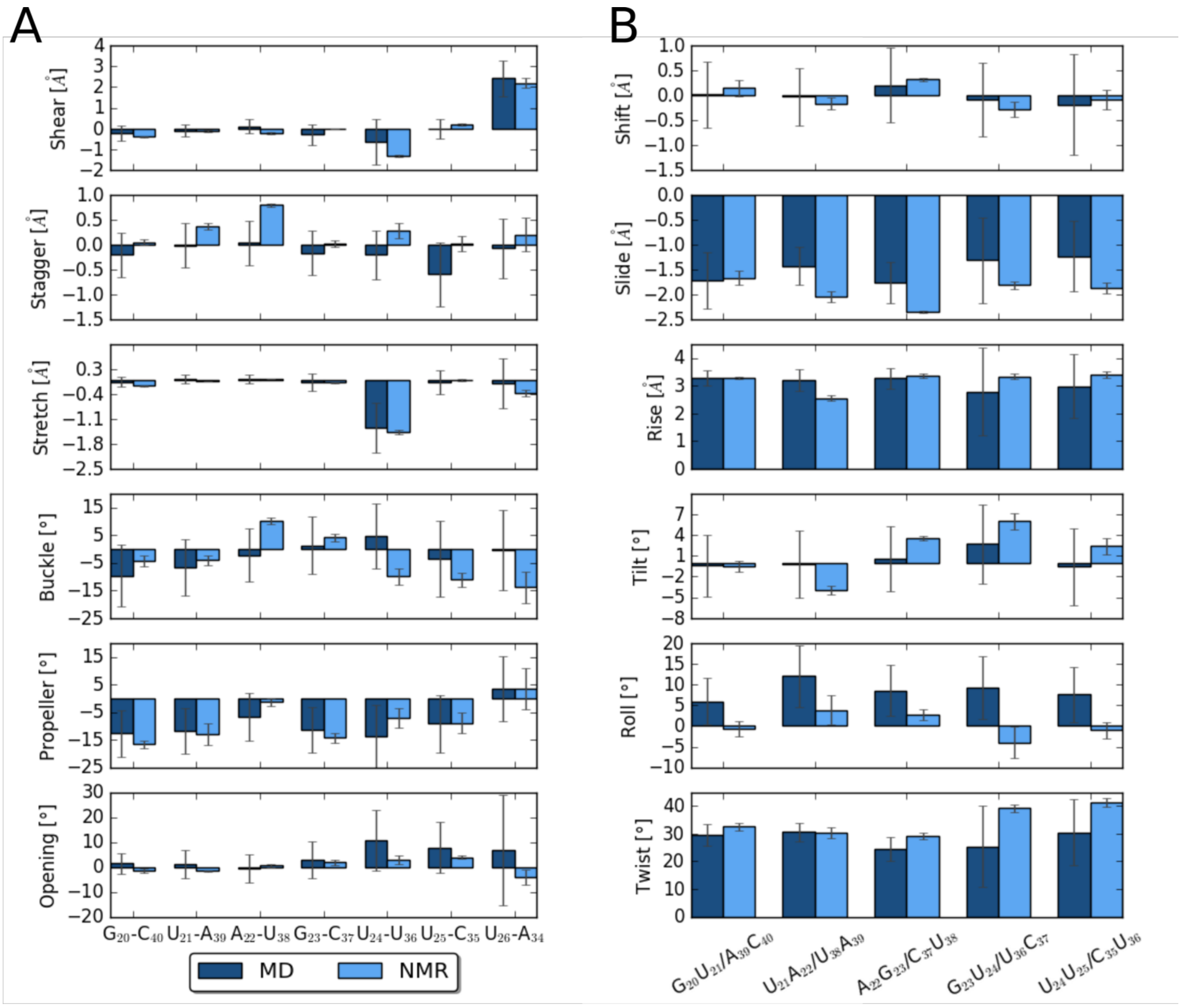

S8 Fig. Base pair (bp) and base pair steps (bps) of pre-miR20b RNA in the free state. (A) bp and (B) bps parameters for base pairs G_{20}-C_{40}, U_{21}-A_{39}, A_{22}-U_{38}, G_{23}-C_{37}, U_{24}-U_{36}, U_{25}-C_{35}, U_{26}-A_{34}, calculated over the entire MD (dark blue) and NMR (light blue) ensembles.
