# Supplementary material for: Molecular basis for the increased affinity of an RNA recognition motif with re-engineered specificity: A molecular dynamics and enhanced sampling simulations study
Source: PLoS Comput Biol. 2018 Dec 6;14(12):e1006642. doi: 10.1371/journal.pcbi.1006642 (PMC6307825; doi:10.1371/journal.pcbi.1006642)
Supplement: S9 Fig — Average Na+ distribution for the (A) free pre-miR20b (Table 1, simulations 2–7) and (B) its complex with the Rbfox protein (Table 1, simulations 8–13), as a function of the distance from the helical axis (R). The results are plotted as molarities as shown by the color bars, with blue to yellow scale indicating increasing values. The vertical white line indicates the radial position of the phosphorus atoms. (PDF) [file pcbi.1006642.s011.pdf]

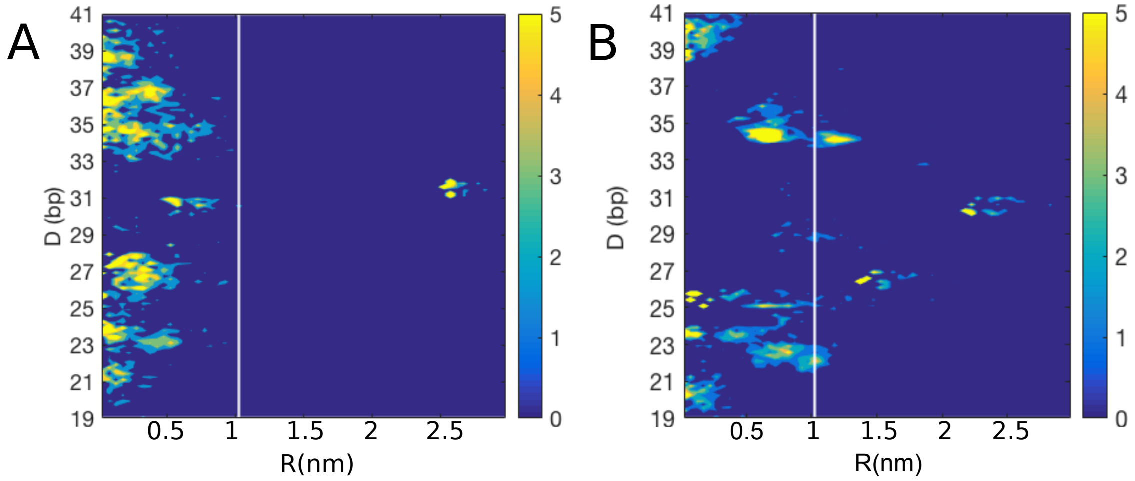

S9 Fig. Average Na$^+$ distribution for the (A) free pre-miR20b (Table 1, simulations 2-7) and (B) its complex with the Rbfox protein (Table 1, simulations 8-13), as a function of the distance from the helical axis (R). The results are plotted as molarities as shown by the color bars, with blue to yellow scale indicating increasing values. The vertical white line indicates the radial position of the phosphorus atoms.
