# Supplementary material for: Molecular basis for the increased affinity of an RNA recognition motif with re-engineered specificity: A molecular dynamics and enhanced sampling simulations study
Source: PLoS Comput Biol. 2018 Dec 6;14(12):e1006642. doi: 10.1371/journal.pcbi.1006642 (PMC6307825; doi:10.1371/journal.pcbi.1006642)
Supplement: S10 Fig — Stacking geometries are described by the center of mass distance d and the angle θ between the planes of the bases and amino acid side chain. Amino acid and nucleobase are considered stacked if d< 0.5 nm and θ <30°. The distributions are calculated for G29-R184, G29-F126, U32-H120 and G33-F160 pairs in the individual unrestrained trajectories (Table 1, sim. 8–13). (PDF) [file pcbi.1006642.s012.pdf]

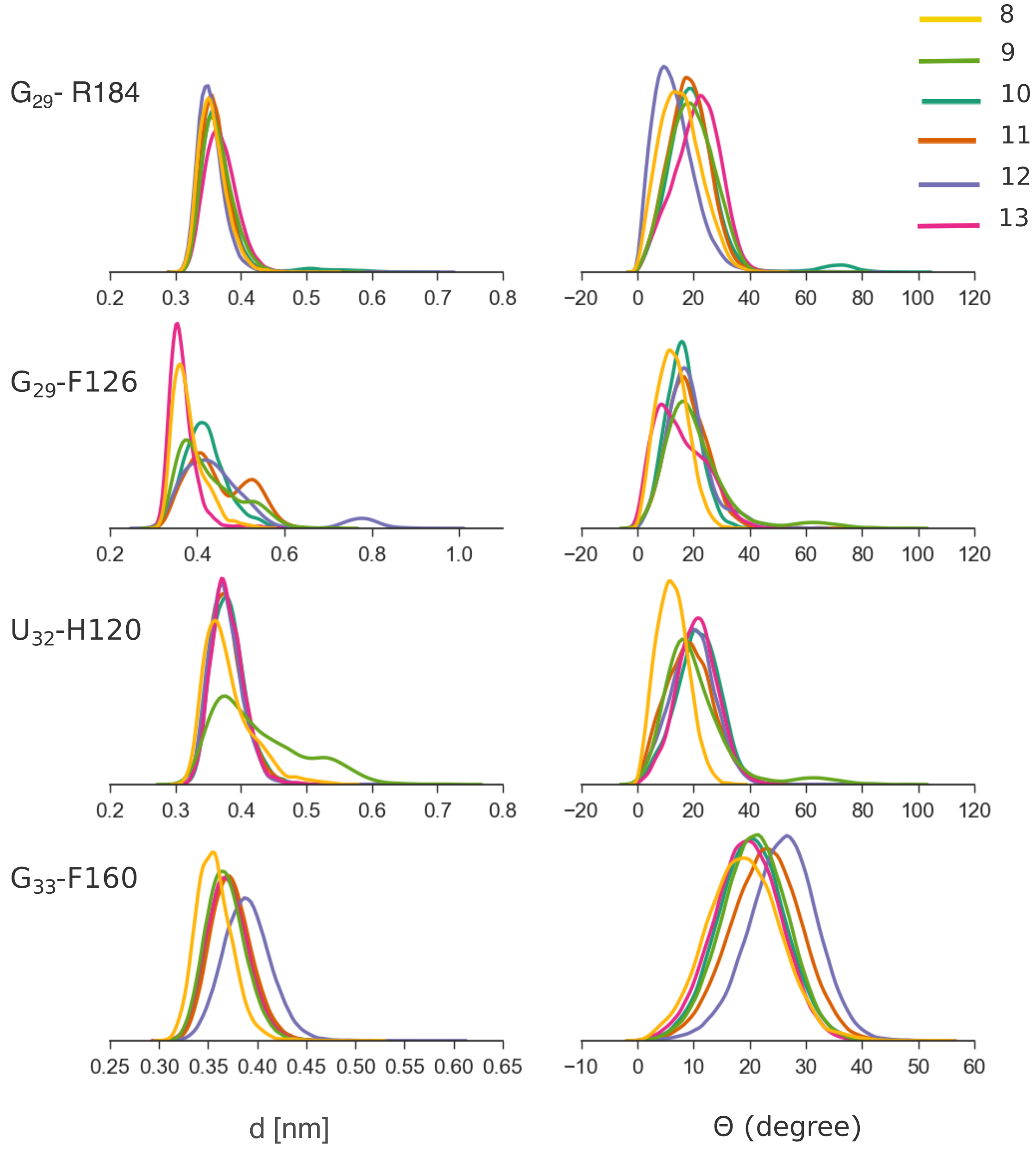

S10 Fig. Stacking interactions in the Rbfox•pre-miR20b complex. Stacking geometries are described by the center of mass distance d and the angle θ between the planes of the bases and amino acid side chain. Amino acid and nucleobase are considered stacked if d< 0.5 nm and θ <30°. The distributions are calculated for G_{29}-R184, G_{29}-F126, U_{32}-H120 and G_{33}-F160 pairs in the individual unrestrained trajectories (Table 1, sim. 8-13).
