# Supplementary material for: Molecular basis for the increased affinity of an RNA recognition motif with re-engineered specificity: A molecular dynamics and enhanced sampling simulations study
Source: PLoS Comput Biol. 2018 Dec 6;14(12):e1006642. doi: 10.1371/journal.pcbi.1006642 (PMC6307825; doi:10.1371/journal.pcbi.1006642)
Supplement: S11 Fig — Calculated and experimentally measured [6] chemical shifts (CS) for pre-miR20b in the free state (A), for pre-miR20b in complex with the Rbfox (B) and for the Rbfox protein (C). The CS have been calculated using the SHIFTX+ [88] for the protein and LARMORD [36] for the RNA. Additional details are reported in the Materials and Methods section. (PDF) [file pcbi.1006642.s013.pdf]

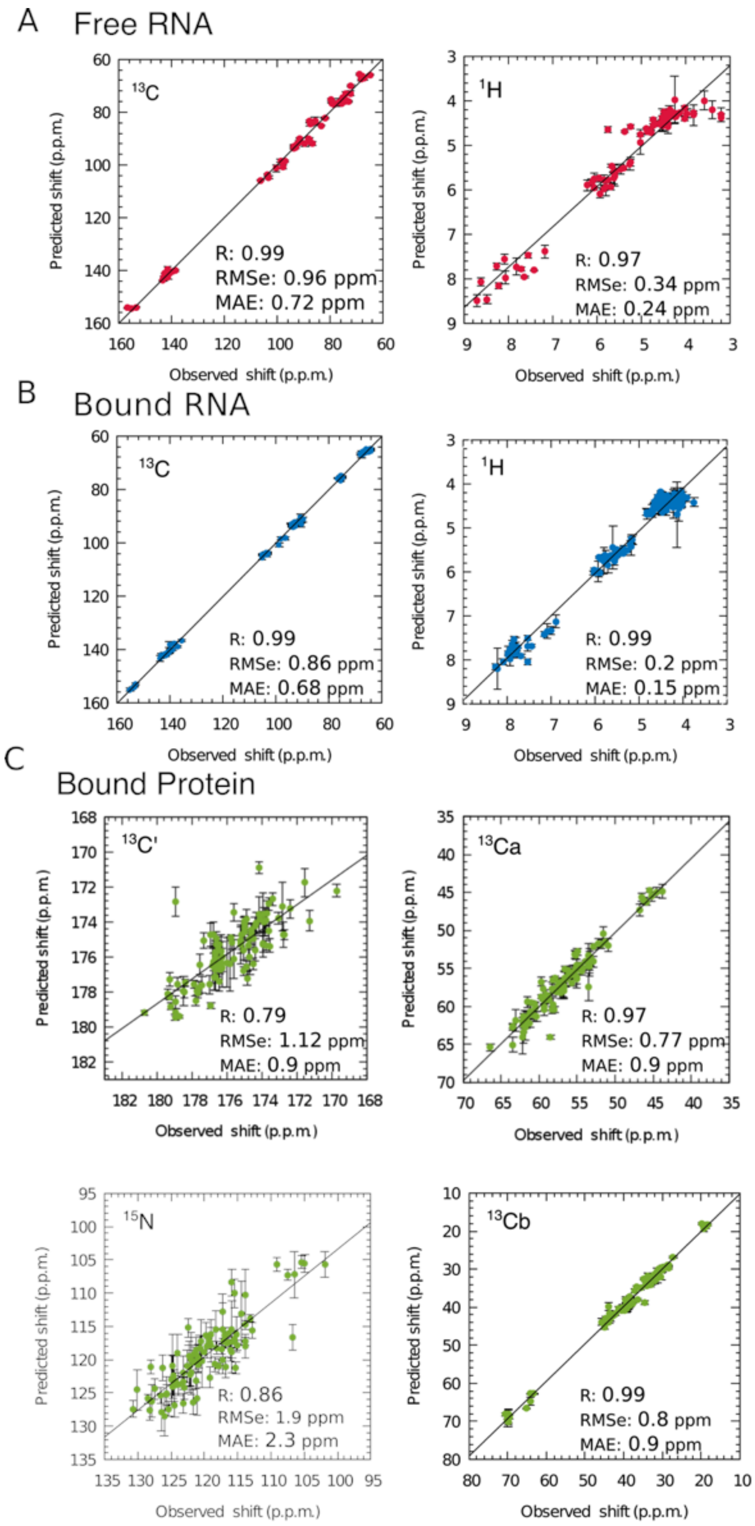

S11 Fig. Calculated and experimentally measured chemical shifts (CS) for pre-miR20b in the free state (A), for pre-miR20b in complex with the Rbfox (B) and for the Rbfox protein (C). The CS have been calculated using the SHIFTX+ for the protein and LARMOR^{D} for the RNA. Additional details are reported in the Materials and Methods section.
