# Supplementary material for: Molecular basis for the increased affinity of an RNA recognition motif with re-engineered specificity: A molecular dynamics and enhanced sampling simulations study
Source: PLoS Comput Biol. 2018 Dec 6;14(12):e1006642. doi: 10.1371/journal.pcbi.1006642 (PMC6307825; doi:10.1371/journal.pcbi.1006642)
Supplement: S12 Fig — The grey square represents the standard deviation. Additional details on the calculations are reported in the Materials and Methods section. (PDF) [file pcbi.1006642.s014.pdf]

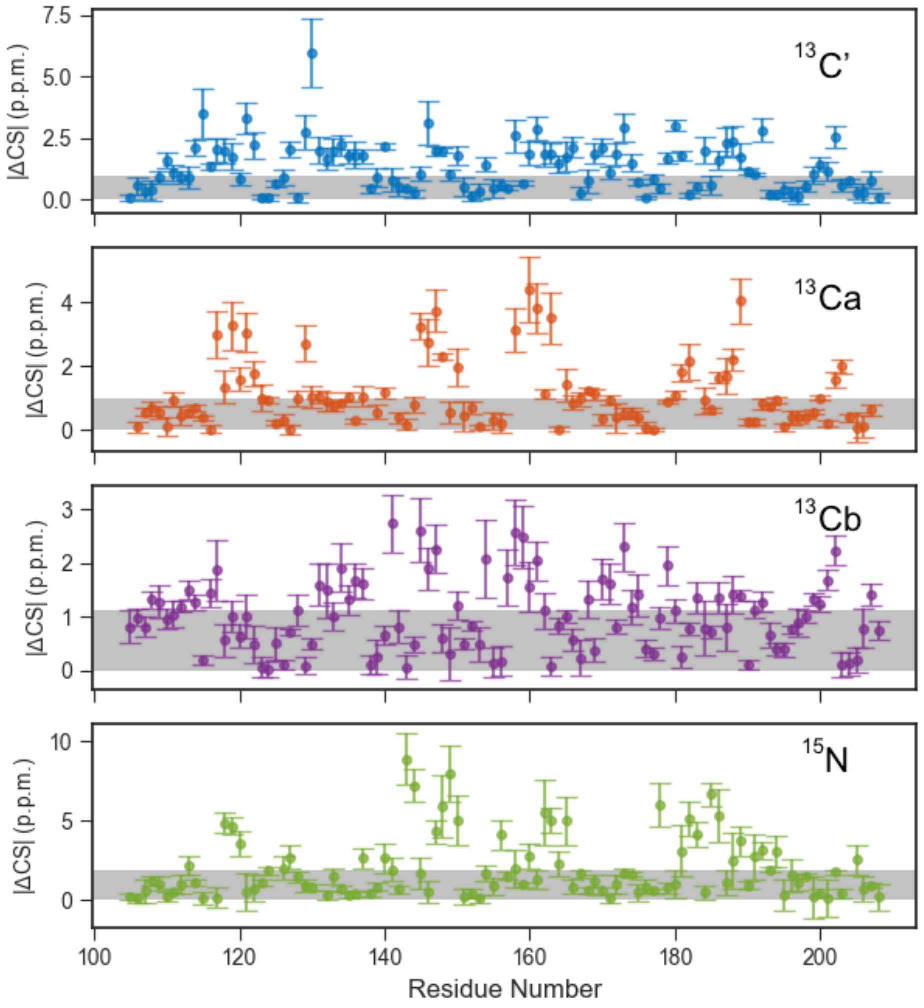

S12 Fig. Comparison between calculated and experimental ^{13}C', ^{13}Ca, ^{13}Cb and ^{15}N CS of Rbfox in the free state and bound to pre-miR20b. The grey square represents the standard deviation. Additional details on the calculations are reported in the Materials and Methods section.
