# Supplementary material for: Molecular basis for the increased affinity of an RNA recognition motif with re-engineered specificity: A molecular dynamics and enhanced sampling simulations study
Source: PLoS Comput Biol. 2018 Dec 6;14(12):e1006642. doi: 10.1371/journal.pcbi.1006642 (PMC6307825; doi:10.1371/journal.pcbi.1006642)
Supplement: S13 Fig — The blue dot represents the calculated average value; the red one corresponds to the experimental value. A similar behaviour is observed in the other simulations performed on the system. (PDF) [file pcbi.1006642.s015.pdf]

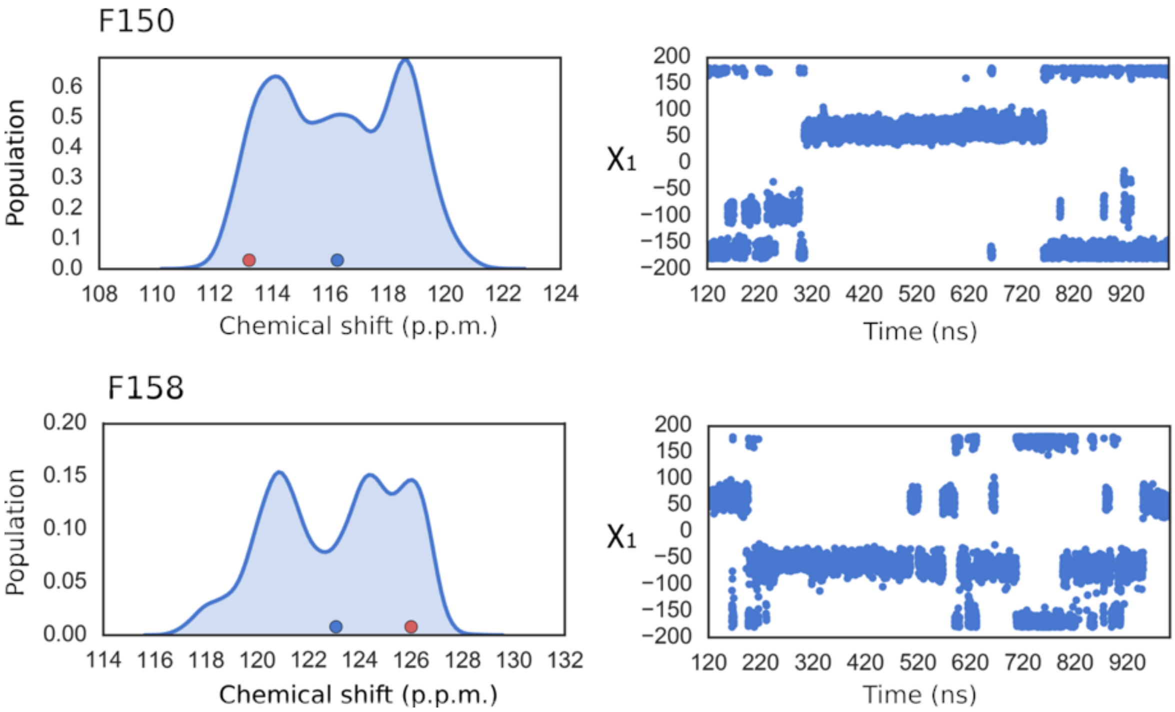

S13 Fig. Distributions of CS predicted by SHIFTX+ for F150 and F158 ^{15}N from the MD simulations of the Rbfox in complex with pre-miR20b (see Materials and Methods for details) and values of the χ_1 angles of the same residues in trajectory 9. The blue dot represents the calculated average value; the red one corresponds to the experimental value. A similar behaviour is observed in the other simulations performed on the system.
