# Supplementary material for: Molecular basis for the increased affinity of an RNA recognition motif with re-engineered specificity: A molecular dynamics and enhanced sampling simulations study
Source: PLoS Comput Biol. 2018 Dec 6;14(12):e1006642. doi: 10.1371/journal.pcbi.1006642 (PMC6307825; doi:10.1371/journal.pcbi.1006642)
Supplement: S14 Fig — Comparison of calculated and experimental chemical shifts for the (A) 13C and (B) 1H atoms of pre-miR20b in the free state (Table 1, sim. 2–7). Representation as in S12 Fig. (PDF) [file pcbi.1006642.s016.pdf]

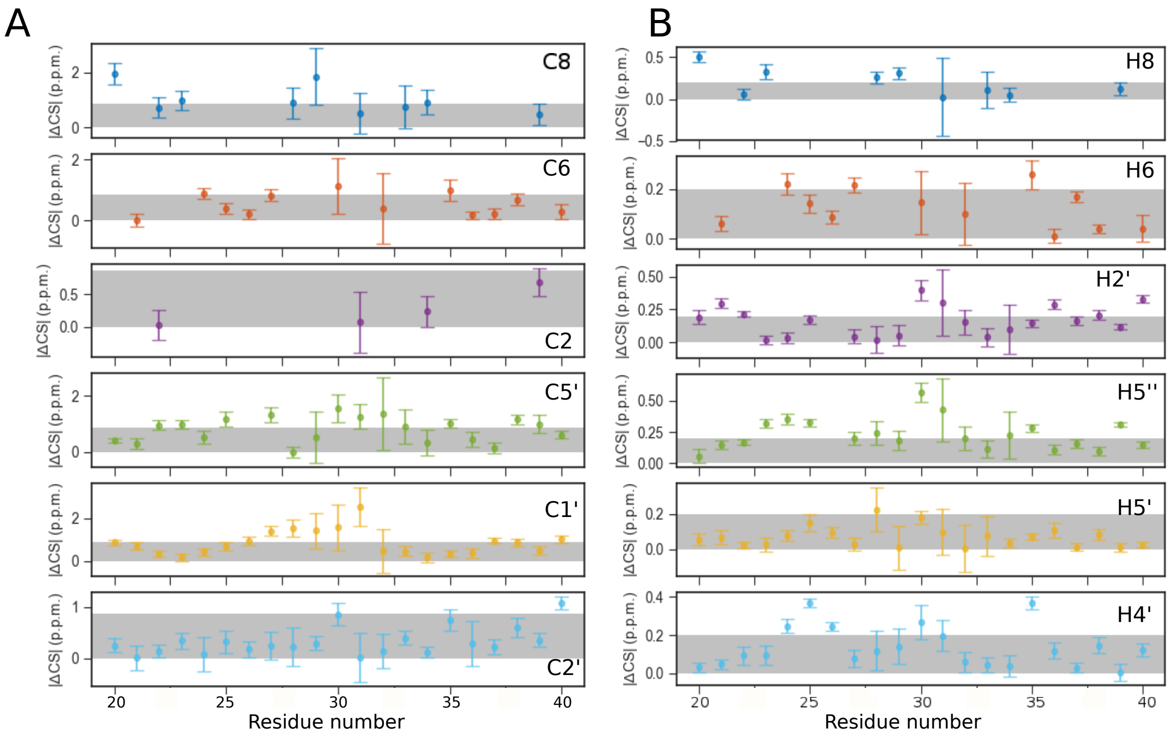

S14 Fig. Comparison of calculated and experimental chemical shifts for the (A) ^{13}C and (B) ^{1}H atoms of pre-miR20b in the free state (Table 1, sim. 2-7). Representation as in S12 Fig.
