# Supplementary material for: Molecular basis for the increased affinity of an RNA recognition motif with re-engineered specificity: A molecular dynamics and enhanced sampling simulations study
Source: PLoS Comput Biol. 2018 Dec 6;14(12):e1006642. doi: 10.1371/journal.pcbi.1006642 (PMC6307825; doi:10.1371/journal.pcbi.1006642)
Supplement: S16 Fig — The distributions are calculated for G29-R184, G29-F126, U32-H120 and C33-F160 pairs in replica exchange (Table 1, sims. 15–16) and unbiased MD (Table 1, sims, 17–18) simulations. (PDF) [file pcbi.1006642.s018.pdf]

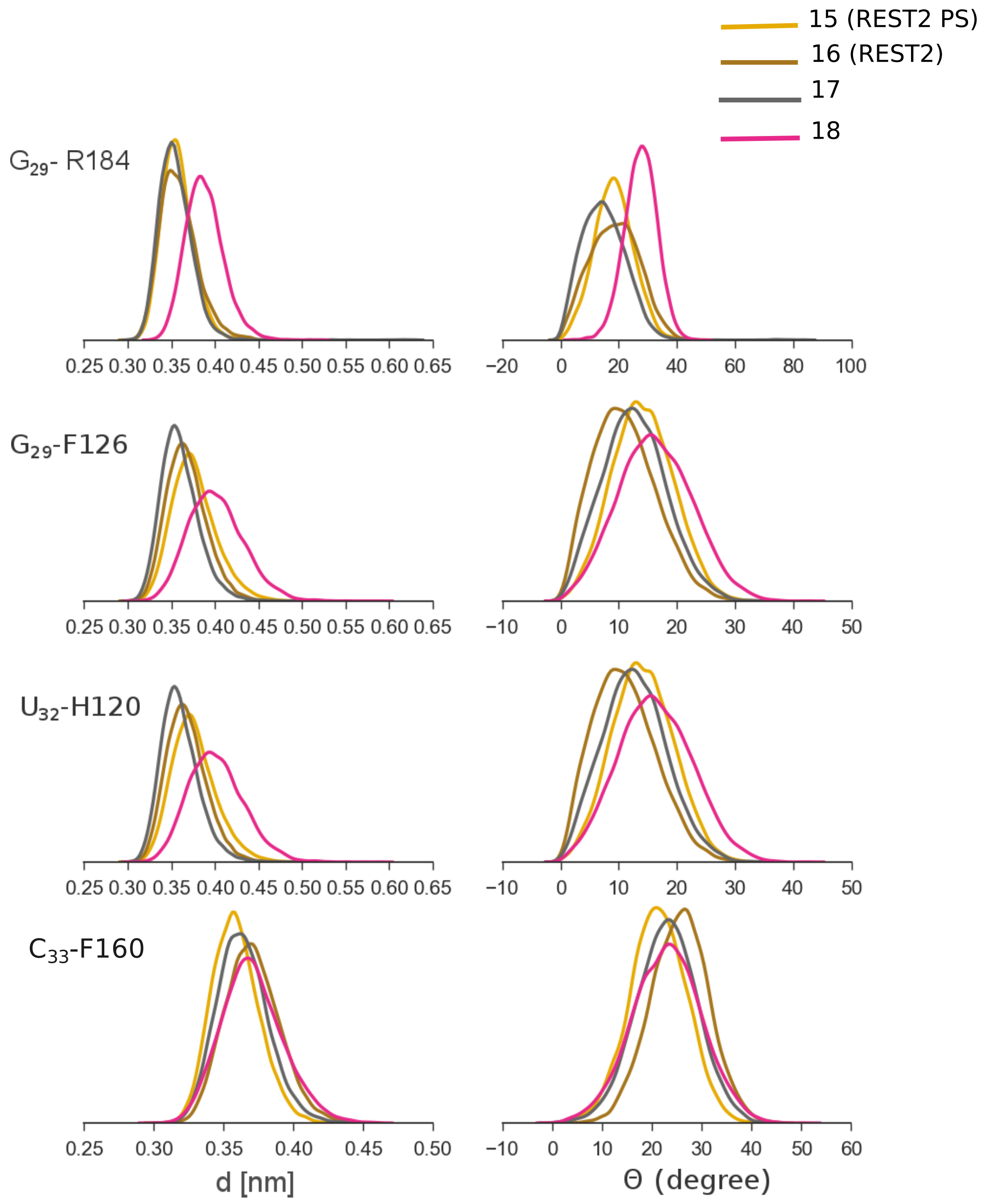

S16 Fig. Stacking interactions in the Rbfox*•pre-miR20b* complex. The distributions are calculated for G_{29}-R184, G_{29}-F126, U_{32}-H120 and C_{33}-F160 pairs in replica exchange (Table 1, sims. 15-16) and unbiased MD (Table 1, sim. 17-18) simulations.
