# Supplementary material for: Molecular basis for the increased affinity of an RNA recognition motif with re-engineered specificity: A molecular dynamics and enhanced sampling simulations study
Source: PLoS Comput Biol. 2018 Dec 6;14(12):e1006642. doi: 10.1371/journal.pcbi.1006642 (PMC6307825; doi:10.1371/journal.pcbi.1006642)
Supplement: S17 Fig — (PDF) [file pcbi.1006642.s019.pdf]

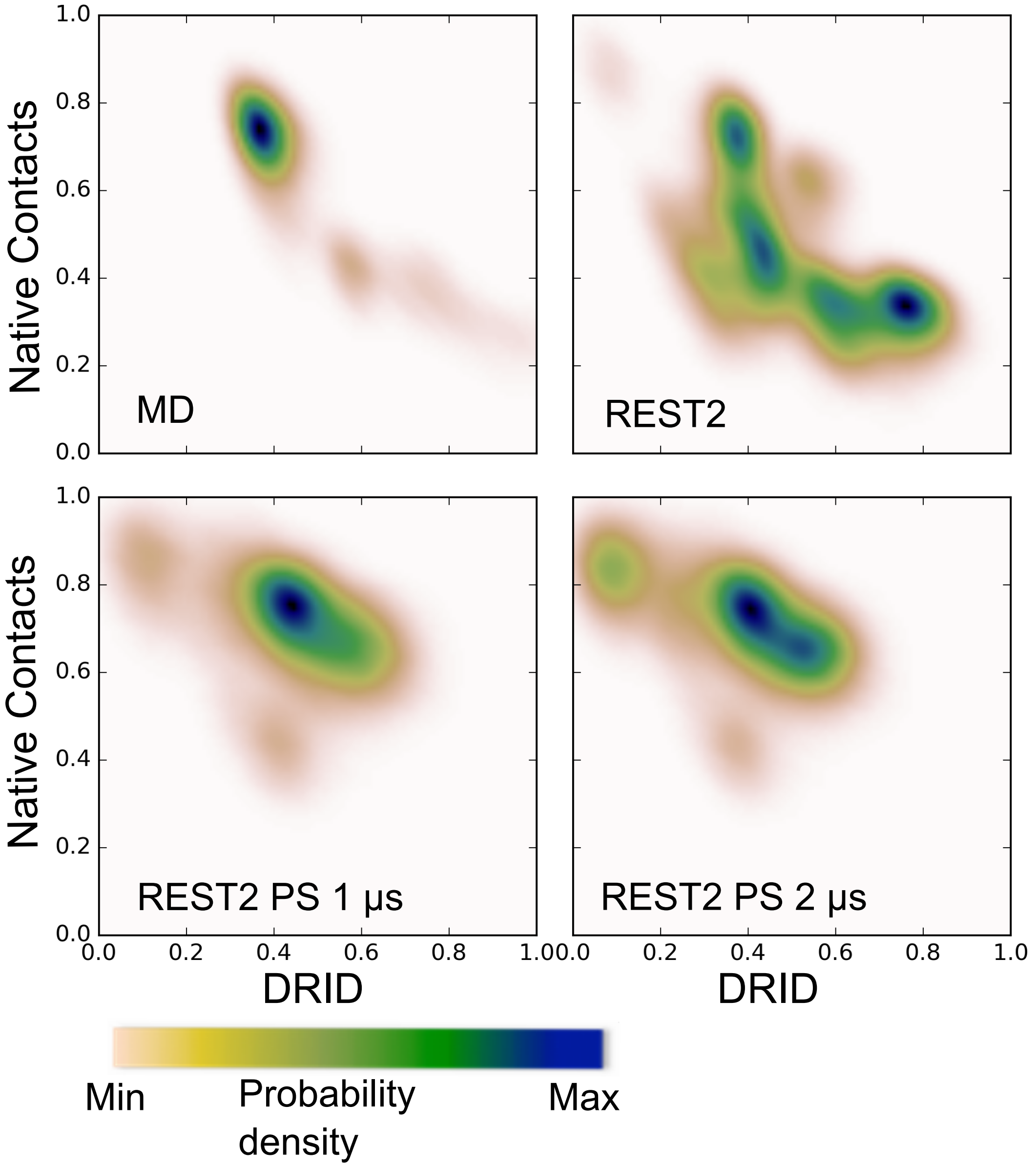

S17 Fig. Probability density of sampling the conformational landscape of the Rbfox*-pre-miR20b* complex in the two-dimensional space of Native Contacts and of DRID (see Method sections for details) for plain MD, conventional (standard) REST2 and REST2 with partial scaling (REST2 PS), respectively.
