# Supplementary material for: Molecular basis for the increased affinity of an RNA recognition motif with re-engineered specificity: A molecular dynamics and enhanced sampling simulations study
Source: PLoS Comput Biol. 2018 Dec 6;14(12):e1006642. doi: 10.1371/journal.pcbi.1006642 (PMC6307825; doi:10.1371/journal.pcbi.1006642)
Supplement: S18 Fig — (A) Top view of the structure in the simulation (Table 1, sim. 23). (B) Details of the stacking interactions of G29 with F126 and R184. (PDF) [file pcbi.1006642.s020.pdf]

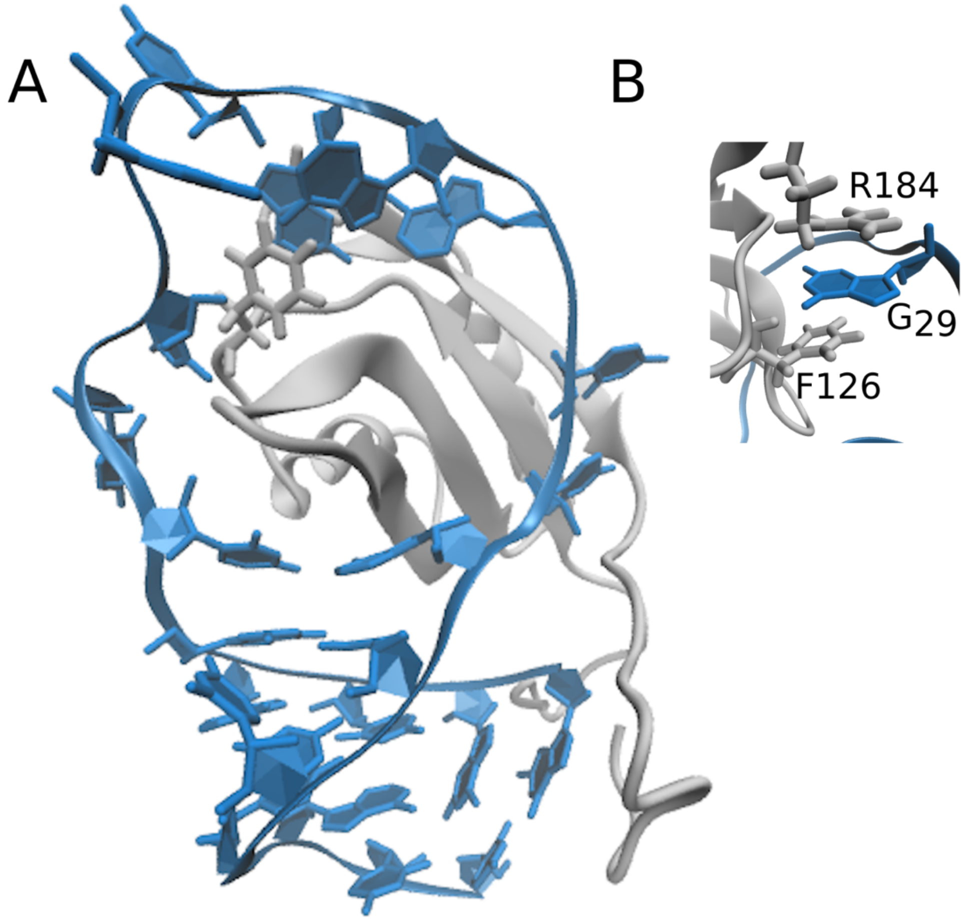

S18 Fig. Rbfox•pre-miR20b* complex. (A) Top view of the structure in the simulation (Table 1, sim. 23). (B) Details of the stacking interactions of G_{29} with F126 and R184.
