# Supplementary material for: Molecular basis for the increased affinity of an RNA recognition motif with re-engineered specificity: A molecular dynamics and enhanced sampling simulations study
Source: PLoS Comput Biol. 2018 Dec 6;14(12):e1006642. doi: 10.1371/journal.pcbi.1006642 (PMC6307825; doi:10.1371/journal.pcbi.1006642)
Supplement: S19 Fig — (A) Top view of the structure in the simulation (Table 1, sim. 24). (B) Details of the H-bond interactions (dashed blue lines) involving G33, R147 and D118. (PDF) [file pcbi.1006642.s021.pdf]

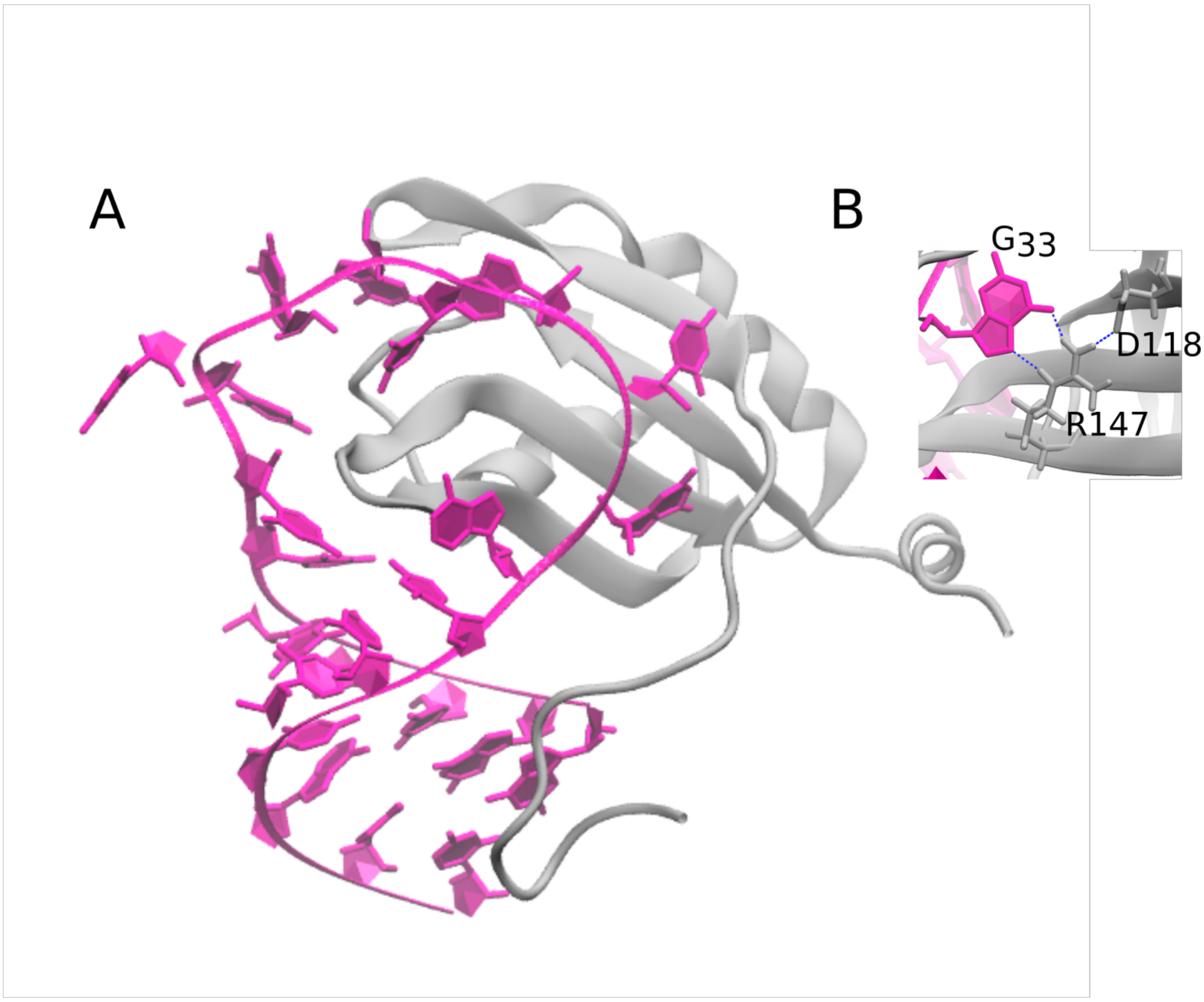

S19 Fig. Rbfox*•pre-miR20b complex. (A) Top view of the structure in the simulation (Table 1, sim. 24).

(B) Details of the H-bond interactions (dashed blue lines) involving G_{33}, R147 and D118.
