# Supplementary material for: Molecular basis for the increased affinity of an RNA recognition motif with re-engineered specificity: A molecular dynamics and enhanced sampling simulations study
Source: PLoS Comput Biol. 2018 Dec 6;14(12):e1006642. doi: 10.1371/journal.pcbi.1006642 (PMC6307825; doi:10.1371/journal.pcbi.1006642)
Supplement: S1 Table — (PDF) [file pcbi.1006642.s022.pdf]

Table S1. Selected region for the "Partial Scaling" REST2 simulation of the Rbfox*□pre-miR20b* complex.

Residues

U_{28}	G153
A_{30}	S154
C_{33}	K155
D117	F160
D144	T162
R146	T192
S150	A193
T151	R194
R152	
