# Supplementary material for: Molecular basis for the increased affinity of an RNA recognition motif with re-engineered specificity: A molecular dynamics and enhanced sampling simulations study
Source: PLoS Comput Biol. 2018 Dec 6;14(12):e1006642. doi: 10.1371/journal.pcbi.1006642 (PMC6307825; doi:10.1371/journal.pcbi.1006642)
Supplement: S2 Table — (PDF) [file pcbi.1006642.s023.pdf]

Table S2. **NOE-upper bounds violations (*v*)** for pre-miR20b in the free state (Table 1, sim. 2-7), Rbfox (Table 1, sim. 1) and Rbfox•pre-miR20b complex (Table 1, sim. 8-13) calculated along all individual MD trajectories and on the "full ensemble" obtained by merging all the unrestrained parts of the initially restrained trajectories.

Structure	Trajectory	*v*<=0.05 nm	0.05 nm <*v*<=0.2 nm	0.2<*v*<=0.3 nm	*v*>=0.3 nm
Rbfox					
	1	13%	1.6%	1%	0.8%
Pre-miR20b					
	2	13%	1.7%	0.7%	0.9%
	3	14%	1.3%	0.5%	/
	4	14%	1.1%	/	/
	5	14%	0.7%	0.2%	/
	6	13%	1.3%	0.9%	0.2%
	7	13%	1.6%	0.5%	0.9%
	Full ensemble	13%	0.4%	/	/
Rbfox•pre-miR20b					
	8	17%	5%	0.2%	1.2%
	9	16%	5%	0.2%	0.8%
	10	16%	5%	0.2%	0.1%
	11	16%	4%	0.15%	0.1%
	12	16%	5%	0.2%	0.1%
	13	16%	5%	0.2%	0.1%
	Full ensemble	15%	4%	0.17%	0.8%
